# The Gut Resistome Atlas in Preterm Infants Enables Prediction of Necrotizing Enterocolitis Onset

**DOI:** 10.1002/advs.202505154

**Published:** 2025-09-30

**Authors:** Shuqin Zeng, Hua Wang, Li Zhang, Shiping Li, Yuan Yuan, Mengting Tian, Yi Qu, Junjie Ying, Meicen Zhou, Yong Hu, Jinglan Huang, Rong Zou, Fengyan Zhao, Xiaojuan Su, Qian Liu, Yan He, Jinxing Feng, Weimin Huang, Ying Luo, Zhemin Zhou, Wei Shen, Dezhi Mu, Shaopu Wang

**Affiliations:** ^1^ Department of Pediatrics West China Second University Hospital Sichuan University Chengdu 610041 China; ^2^ Key Laboratory of Birth Defects and Related Diseases of Women and Children (Sichuan University) Ministry of Education West China Second University Hospital Sichuan University Chengdu 610041 China; ^3^ Guangzhou Women and Children's Medical Center Guangzhou 510600 China; ^4^ Microbiome Medicine Center Department of Laboratory Medicine Zhujiang Hospital Southern Medical University Guangzhou 510280 China; ^5^ Guangdong Provincial Clinical Research Center for Laboratory Medicine Guangzhou 510033 China; ^6^ Shenzhen Children's Hospital Shenzhen 518026 China; ^7^ Foshan Women and Children Hospital Foshan 528000 China; ^8^ Key Laboratory of Alkene‐Carbon Fibres‐Based Technology & Application for Detection of Major Infectious Diseases MOE Key Laboratory of Geriatric Diseases and Immunology Cancer Institute Suzhou Medical College Soochow University Suzhou 215123 China; ^9^ Department of Neonatology Nanfang Hospital Southern Medical University Guangzhou 510515 China

**Keywords:** biomarkers, gut resistome, metagenomics, NEC, preterm infants

## Abstract

The accelerating threat from antimicrobial resistance (AMR) has become a global health issue. The properties of AMR in the gut microbiome of preterm infants and its clinical relevance with necrotizing enterocolitis (NEC) remain unknown. In‐depth integrative analyses of 5,684 gut metagenomes are performed to build an AMR genes (ARGs) landscape. A subset of 107 preterm infants who developed NEC is sampled to examine the trajectory and predictive potential of ARGs preceding NEC onset. The variation and core set of ARGs, their higher burden and diversity, and potential ARGs‐enriched gut bacteria in preterm infants compared to full‐term infants are comprehensively discovered, reflecting a strain shift in genomic functions. Moreover, the gut resistome converged over 9 days before NEC onset is observed, which is driven by 24 ARGs. Machine learning analysis reveals potential usage of the gut resistome as an indicator for predicting NEC onset in an external validation preterm birth cohort (the area under the receiver operating characteristic curve, AU‐ROC = 0.823), which is significantly higher than that based on the bacterial species (AU‐ROC = 0.727). Overall, the findings can be referenced to mitigate the burden and spread of ARGs, and specific ARGs have potential for disease risk stratification to improve clinical management.

## Graphical abstract

The gut resistome of preterm infants and its clinical relevance remain poorly understood. This study performs in‐depth integrative analyses of 5,684 gut metagenomes from preterm infants, uncovering distinct features that diverge from term infants. Notably, the gut resistome of preterm infants converges 9 days before necrotizing enterocolitis onset, highlighting its potential for early disease risk prediction.

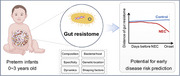

## 1 Introduction

The prevalence of preterm birth, which refers to a live birth at < 37 gestational weeks, is estimated at 11% of all births worldwide (≈15 million infants) and has become a major challenge for infant health during hospitalization, as well as causing health problems later in life.^[^ ^1^ ^]^ Variations in the risk associated with preterm birth depend markedly on gestational age; that is, a lower gestational age is associated with an increased incidence of complications. Recent evidence suggests that abnormal development of the gut microbiome in preterm infants is closely associated with a wide range of diseases, including necrotizing enterocolitis (NEC) and late­onset sepsis, highlighting the central role of the gut microbiome in human health.^[^ ^2^, ^3^, ^4^, ^5^, ^6^ ^]^

Antibiotics are commonly prescribed to preterm infants during the first few days of life in the neonatal intensive care unit (NICU) to prevent the occurrence of possible infections and related morbidity and mortality.^[^ ^7^ ^]^ It has been reported that 79% and 87% of preterm infants born with very low birth weight or extremely low birth weight in the United States are treated with antibiotics in the first three days of life,^[^ ^8^ ^]^ and 82% of very preterm infants in China receive antibiotics immediately after birth.^[^ ^9^ ^]^ Although antibiotics are designed to eliminate pathogens, their broad‐spectrum activity exerts selective pressure on commensal microbes due to evolutionarily conserved molecular targets shared across bacterial species, which results in dysbiosis of the gut microbiome and enrichment of genes for antimicrobial resistance (AMR).^[^ ^10^ ^]^ This phenomenon is particularly pronounced in preterm infants, who experience frequent exposures to antibiotics and prolonged hospitalization.

The human gut is a major reservoir for host‐associated bacteria with AMR genes (ARGs, collectively termed the resistome). While the gut microbiome and resistome are inherently interconnected, current metagenomic studies predominantly focus on the overall gut microbiome in health and disease.^[^ ^11^, ^12^ ^]^ Our knowledge regarding the compositional, developmental properties, and the potential clinical implications of the gut resistome in diverse populations of preterm infants remains largely lagging behind. Therefore, increased research efforts specifically dedicated to exploring the gut resistome in preterm infants are clearly warranted.^[^ ^13^, ^14^, ^15^, ^16^ ^]^

The NEC, a serious intestinal inflammation, is a leading cause of death and morbidity in preterm infants; however, its etiopathology is still poorly understood.^[^ ^17^ ^]^ It remains uncertain whether pre‐onset bacterial diversity truly reflects the risk of NEC onset, as differences in bacterial diversity between infants with or without NEC are reported to be inconsistent.^[^ ^18^, ^19^, ^20^, ^21^ ^]^ Some specific potential biomarkers from the gut bacteriome, maternal milk, and gut virome before NEC onset have been gradually reported;^[^ ^4^, ^19^, ^22^ ^]^ these biomarkers, once they have been validated and generalized in multi‐center studies, may prove to be beneficial in the early detection of disease onset and the development of microbiome‐based therapeutics. However, how the gut resistome of preterm infants or any specific ARGs varies during NEC progression, and to what extent they can be used as a proxy to predict disease onset, has yet to be investigated. Notably, evidence from low‐ and middle‐income countries in Africa and South Asia suggests that neonates or those whose mothers harbor beta‐lactamase genes in their gut microbiome are more likely to develop sepsis in early life.^[^ ^23^ ^]^

To advance our understanding of the human gut resistome in early life,^[^ ^13^, ^14^, ^15^, ^16^, ^24^ ^]^ we developed a comprehensive landscape of the gut resistome in preterm infants within the first three years of life by applying both a read‐based and assembly‐based approach in examining 5684 gut metagenomes. We first fully profiled the type, abundance, and prevalence of the gut resistome (i.e., ARG types and subtypes from the read‐based approach, and ARGs and drug classes from the assembly‐based approach) across cohorts of preterm infants. Next, we determined the developmental trajectory, shaping factors, the potential bacterial host taxa, and the genetic location of the gut resistome in preterm infants. Subsequently, the shared and unique features of the gut resistome in preterm infants were identified in comparison to those of term infants. Based on longitudinal sampling from preterm infants who subsequently developed NEC, we observed accelerated convergence of the gut resistome before NEC ensued and further trained the model to predict NEC onset with an external preterm birth cohort that had been newly recruited.

## 2 Results

### 2.1 Genomic Composition and Variation of the Gut Resistome in Preterm Infants

To unravel the landscape of the gut resistome in preterm infants in the first three years of life, we conducted an integrative analysis of 5684 stool samples, of which 179 were newly collected and 5505 were available in a public repository (Figure S1 and Table S1, Supporting Information). These samples were analyzed from 682 preterm infants with a median gestational age of 28.0 weeks (interquartile range (IQR) = 25.9–31.0 weeks, except eight preterm infants without this information available from NguyenM_2021) aging from birth to three years old (median = 25 days, IQR = 15–45 days; Figure S2a, Supporting Information) spanning 19 cohorts, geographically distributed in seven countries over three continents, i.e., the United States (*n* = 13 cohorts), the United Kingdom and Finland (*n* = 2 for each), and one cohort each from China, Russia, Estonia, and Luxembourg. Another cohort from Vatanen et al.^[^ ^25^ ^]^ consisted of samples from three countries. All cohorts, excepting the cohort from Rose et al.,^[^ ^26^ ^]^ were sampled longitudinally (median = 6, IQR = 4–11 samples per infant). Additional host information, including delivery mode, feeding pattern, birth weight, and sex, was curated manually (Table S1, Supporting Information).

**Figure 1:**
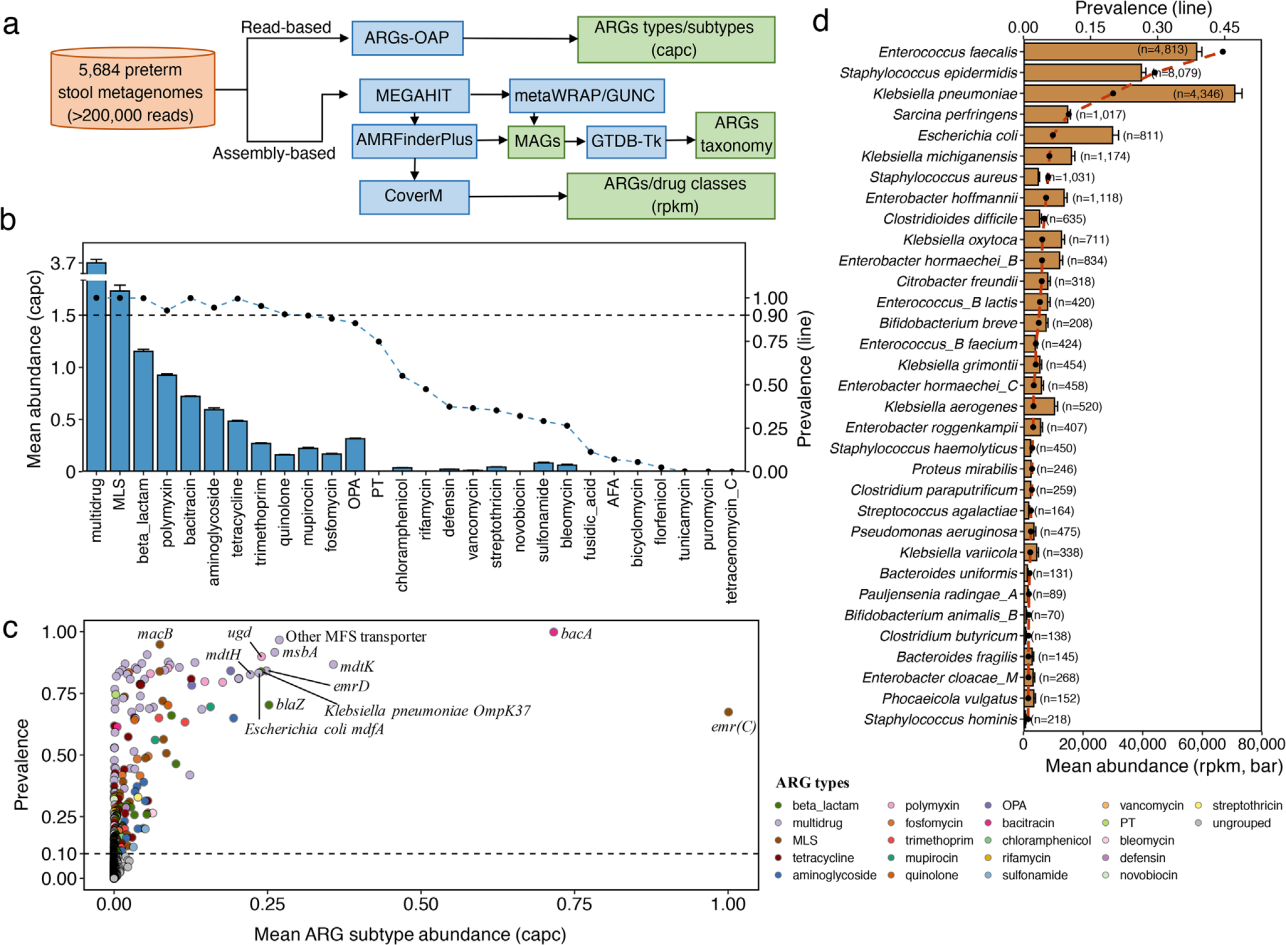
Generating the landscape of gut resistome in preterm infants. a) The schematic overview of the pipeline to explore gut resistome from 5684 new and public gut samples using both read‐based and assembly‐based approaches. b) The prevalence (line) and mean abundance (bar) of ARG types across all samples from the read‐based approach. AFA = antibacterial fatty acid, MLS = macrolide‐lincosamide‐streptogramin, PT = pleuromutilin‐tiamulin, OPA = other peptide antibiotics. c) The prevalence and mean abundance of ARG subtypes from the read‐based approach. The colors indicate the types of ARG subtypes if their prevalence was >10%, and all other ARG subtypes were colored in grey. MFS = major facilitator superfamily. d) The prevalence (line) and mean abundance (bar) of ARG bacterial carriers at the species level based on the assembly approach. Only species with a prevalence > 1% among samples are plotted, and the number in parentheses indicates the number of ARG ORFs harbored by that species. The values in the bar plots indicate the mean abundance of ARGs carried by the corresponding species. All the values in the bar plots are presented as mean ± SE.

Given the advantages and disadvantages of the read‐based (having more sensitivity to ARGs harbored by low‐abundance microorganisms in complex microbial communities that may be missed by the assembly‐based approach) and the assembly‐based (enabling the investigation of the genomic context of ARGs, such as taxonomic assignment and mobility) approaches,^[^ ^27^ ^]^ we simultaneously applied these two independent algorithms in our pipeline to explore the gut resistome in preterm infants (**Figure** **1**a). As expected, at the gene level, a higher number of ARG subtypes (median = 121; IQR = 91–155) was detected than assembled ARGs (referred to as aARGs) (median = 13; IQR = 8–18), which however were highly correlated (Pearson's *r* = 0.43, *P* < 0.001, Figure S2b, Supporting Information) on a per‐sample basis.

In total, 2033 ARG subtypes belonging to 28 types (out of 30 types in total from SARG database^[^ ^28^ ^]^) were annotated in at least one stool sample, with a median prevalence of 51.3% (IQR = 22.7–93.1%) and mean abundance from 9.59 × 10^−8^ to 3.65 capc (copies of ARGs per prokaryote's cell) across all samples for types (Table S2, Supporting Information). Each sample contained ≥ 2 ARG types (median = 15, IQR = 14–17), and types of multidrug and macrolide‐lincosamide‐streptogramin (MLS) were present in almost all samples (*n* = 5684 and 5680, respectively), followed by the other seven types with > 90% prevalence; these nine types accounted for a median of 91.0% (IQR = 87.7%–95.0%) of the total ARG burden (Figure 1b). The mean abundance of ARGs showing multidrug resistance was found to be ≥ 2‐fold higher than that of other ARG types. Regarding the genotypes of ARGs, 13 subtypes were present with a mean abundance > 0.2 capc at a prevalence from 67.4% to 99.8%, which accounted for a median of 41.6% (IQR = 30.4%–48.7%) of the total ARG burden (Figure 1c). When classifying ARG subtypes into types, beta‐lactam had the highest proportion of subtypes (63.7% with 1295 subtypes), followed by aminoglycoside (*n* = 123), multidrug (*n* = 111), MLS (*n* = 105), quinolone (*n* = 104), and other ARG types with 1–65 subtypes.

Based on the assembly approach, 80526 ARG open reading frames (ORFs) were extracted and annotated to 623 aARGs, conferring resistance to 54 drug classes (median = 13, IQR = 8–18 on a per‐sample basis) (Table S3, Supporting Information). 55.9% of aARGs overlapped with ARG subtypes, and their prevalence among samples was highly correlated (Pearson's r = 0.77, *P* < 0.001, Figure S2c, Supporting Information). A total of 33 aARGs were prevalent in > 10% of the samples, with *tet(M)*, *oqxA*, *Isa(A)*, *blaI*, *oqxB*, and *blaR1* being the most common with > 40% prevalence (Figure S2d and Table S4, Supporting Information). Although there were great variations in terms of the prevalence of drug classes, from 0.02% for virginiamycin M to 78.9% for tetracycline, there were 13 drug classes with > 50% prevalence that accounted for a median of 85.4% (IQR = 70.0–97.2%) of the total ARG burden, included tetracycline, beta‐lactam, erythromycin, streptogramin, streptogramin B, fosfomycin, clindamycin, lincosamide, quinolone, cephalosporin, phenicol, kanamycin, and tylosin (Figure S2e and Table S4, Supporting Information).

Additionally, we found that a high proportion of ARG subtypes (77.5% and 52.1%) and aARGs (92% and 77%) were specialists that were rarely detected, with a prevalence of < 5% and < 1% of samples, respectively. A total of 182 ARG subtypes were exclusively present in one sample; and when stratifying these exclusive subtypes into each cohort, we found that the majority of exclusive subtypes were from newly sequenced samples (*n* = 95), ThanertR_2024 (*n* = 24), MasiAC_2021 (*n* = 15), and LouYC_2021 (*n* = 15) (Figure S2g, Supporting Information). Similarly, among exclusive aARGs (*n* = 93), MasiAC_2021 (*n* = 16), LouYC_2021 (*n* = 14), and ThanertR_2024 (*n* = 12) had a comparable number of exclusive genes to those found in the newly sequenced samples (*n* = 19) (Figure S2f, Supporting Information). The number of exclusive ARG subtypes or aARGs did not correlate with the sequencing depth of each sample (Figure S2g, Supporting Information), reflecting variations in the gut resistome of preterm infants.

### 2.2 Potential Bacterial Host Taxa and Genetic Location of ARGs in Preterm Infants

To predict which potential taxa primarily host aARGs in the gut microbiome of preterm infants, we binned contigs into metagenome‐assembled genomes (MAGs), and the potential host of aARGs was labeled as the taxonomic assignment of MAGs on which their corresponding ORFs in each sample were located. However, 53.9% (*n* = 43408) of the 80526 ARG ORFs were not binned into any MAGs and thus failed to link to any microbial organisms. All binned aARGs were potentially hosted by bacteria, including 9 known phyla, 11 known classes, 29 known orders, 57 known families, 167 known genera, and 476 known species with 36931 ARG ORFs from the 525 aARGs (Figure S3, Supporting Information). The predominant genera potentially harboring high numbers of ARG ORFs were: *Staphylococcus*, *Klebsiella*, *Enterococcus*, *Streptococcus*, *Enterobacter*, and *Escherichia*. At the species level, apart from members of ESKAPEE (*Enterococcus faecium*, *Staphylococcus aureus*, *Klebsiella pneumoniae*, *Pseudomonas aeruginosa*, *Enterobacter* spp. and *Escherichia coli*), there were 23 additional species harboring aARGs with > 1% prevalence, including species of *Klebsiella (*e.g., *K. michiganensis*, *K. oxytoca*, *K. grimontii*, *K. aerogenes*, and *K. variicola)*, *Staphylococcus* (e.g., *S. epidermidis*, *S. haemolyticus*, and *S. hominis*), *Enterococcus faecalis*, and *Clostridium* (e.g., *C. paraputrificum* and *C. butyricum*) (Figure 1d). *E. faecalis*, *S. epidermidis*, and *K. pneumoniae* were the most prevalent and abundant ARG‐harboring species, which is an addition to previous findings.^[^ ^14^, ^15^ ^]^

As for the genetic location (chromosome or plasmid) of ARGs in the microbial host cell, we found that 27.2% (*n* = 21901) of ARG ORFs covering 191 aARGs could be classified as plasmid‐borne by both tools (Figure S4a, Supporting Information). Additionally, the proportion of plasmid‐borne ORFs among the total unbinned ARG ORFs was higher (39.7% vs 12.5%, Chi‐squared test, *P* < 0.001) than that of binned ARG ORFs, which is consistent with the fact that contigs containing mobile DNA are highly likely to be left unbinned.^[^ ^29^ ^]^ Moreover, to address the diverse distribution of plasmid‐borne aARGs among bacterial species in the gut of preterm infants, we obtained 4648 ORFs of plasmid‐borne aARGs with potential taxonomic assignments and clustered them based on their nucleotide identity, that is, > 99% identity over 90% coverage of ORFs,^[^ ^30^ ^]^ resulting in 277 clusters. The clusters containing ARG ORFs that were assigned to multiple potential species (≥ 2) were labelled as “multi‐species”. When the clusters simultaneously contained ARG ORFs taxonomically assigned and those without (10 of 4648 ORFs without taxa at the species level), the clusters were also labelled as “multi‐species”, speculating that the taxonomically unassigned ARG ORFs were harbored by the different species compared to other ARG ORFs within this cluster. As a result, 122 multi‐species clusters were detected, all of which exclusively contained ARG ORFs with taxonomically assignments, belonging to 112 known species (Figure S4b and Table S5, Supporting Information). Looking into these 112 species, we found that *S. epidermidis* was widely involved in hosting aARGs from 47 clusters, sharing a high number of clusters with *E. faecalis* (*n* = 32), *S. aureus* (*n* = 26), *S. haemolyticus* (*n* = 20), and *S. hominis* (*n* = 15). At the gene level, we found that cluster 150 coding *erm(C)* was the most diverse group with 29 species, followed by cluster 127 coding *aph(3′')‐Ib* with 23 species, and cluster 118 coding *aph(6)‐Id* with 22 species (Table S5, Supporting Information).

### 2.3 Gut Resistome of Preterm Infants Hosting Specific ARGs Compared to Term Infants

While differential antibiotic exposure in preterm versus term infants is hypothesized to drive distinct gut resistome profiles, critical knowledge gaps persist regarding systematic comparisons between the two groups of populations. To broaden our understanding of specific ARG features that are present in preterm infants, we analyzed 1619 stool samples longitudinally (median = 4, IQR = 2–6 samples per infant) collected from 359 infants born term from eight studies that were included in the analysis for preterm infants (Table S6, Supporting Information). The gut resistome of term infants was also characterized using read‐based and assembly‐based approaches as applied to preterm infants for comparable comparisons.

In term infants, 1521 ARG subtypes corresponding to 26 types and 375 aARGs resistant to 45 drug classes were respectively detected, differing in prevalence and abundance. The median prevalence of ARG types was 78.2% (IQR = 24.8–98.5%) with mean abundance from 4.62 × 10^−6^ to 0.71 capc across samples (Table S7, Supporting Information). Multidrug, tetracycline, MLS, and bacitracin ARG types were present in all samples (*n* = 1619), followed by polymyxin, beta‐lactam, mupirocin, pleuromutilin tiamulin (PT), and aminoglycoside, which were present in > 90% of samples. Notably, the PT type was found in > 96.7% of samples, but with an extremely low average abundance of 4.06 × 10^−3^ capc (Figure S5a, Supporting Information). Using the assembly approach, 15914 ARG ORFs were annotated to 375 aARGs among samples, with a median of seven aARGs (IQR = 4–13) per sample. There were only three ARG drug classes with a prevalence of > 50% in the gut resistome of term infants: tetracycline, cephalosporin, and beta‐lactam (Figure S5b and Table S8, Supporting Information).

**Figure 2:**
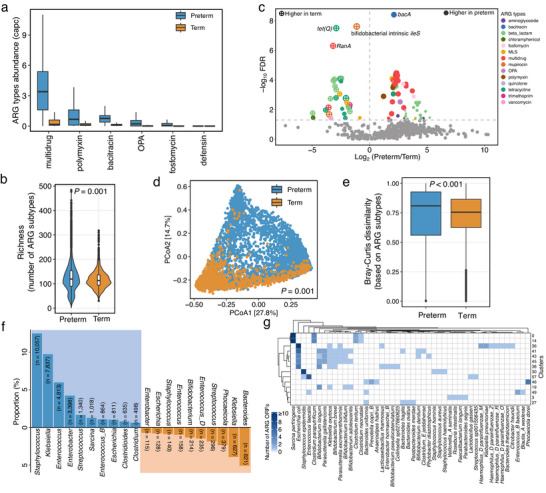
Comparisons of gut resistome between preterm and term infants. a) Preterm infants harbored higher abundances (linear mixed‐effect model, FDR < 0.05) of six ARG types than those of term infants. All values are presented as mean ± SE. b) Richness of ARG subtypes in preterm infants was higher (linear mixed‐effect model, *P* = 0.001) than that of term infants. c) Preterm infants harbored 54 subtypes with higher abundances and 29 subtypes with lower abundances than those of term infants (linear mixed‐effect model, FDR < 0.05). The horizontal dashed line indicates the threshold of significance (‐log_10_(0.05)). Only ARG subtypes with a prevalence >5% in either preterm or term infants were analyzed. d) Principal coordinate analysis (PCoA) based on Bray‐Curtis distances of ARG subtypes abundances from samples of preterm and term infants (PERMANOVA, *P* = 0.001). e) The mean Bray‐Curtis distances calculated from ARG subtypes between samples from preterm infants were lower (two‐sided Wilcoxon rank‐sum test, *P* < 0.0001) than those of term infants. The box plots show the interquartile range (IQR), with a horizontal line as the median, points in the box as the mean, whiskers as the range of the data (up to 1.5× IQR), and points beyond the whiskers as outliers. f) Top ten genera harboring the highest number of ARGs (assembly‐based) from preterm or term infants. g) Distribution of ARG ORFs among bacterial species from clusters in term infants. Only clusters containing ≥ 4 species are plotted. The color gradient indicates the number of ORFs from the species of the corresponding column.

When compared to term infants, the gut microbiome of preterm infants harbored a higher burden of ARG types (1.77 vs 9.34 on average; linear mixed‐effect model, *P* < 0.001), which is consistent with the findings of a previous study.^[^ ^24^ ^]^ More specifically, six ARG types, including multidrug, polymyxin, bacitracin, OPA, fosfomycin, and defensin, exhibited higher abundances (linear mixed‐effect model, FDR < 0.05); whereas no ARG types were present with higher abundances in term infants (**Figure** **2**a). In a higher resolution analysis of ARG subtypes, we found that preterm infants had a higher (linear mixed‐effect model, *P* = 0.001) alpha diversity reflected by the number of subtypes as well as the corresponding Shannon diversity (linear mixed‐effect model, *P* < 0.001; Figure 2b; Figure S5c, Supporting Information). A total of 558 and 46 subtypes were present exclusively in preterm and term infants, respectively. After filtering subtypes with a prevalence >5% in preterm or term infants, 54 subtypes were significantly (linear mixed‐effect model, FDR < 0.05) enriched in preterm infants, whereas 29 subtypes were enriched (linear mixed‐effect model, FDR < 0.05) in term infants (Figure 2c; Table S9, Supporting Information). We further calculated the Bray‐Curtis distances between samples from the abundance of ARG subtypes and found that the gut resistome of preterm or term infants was clustered separately, with a statistically significant difference (PERMANOVA, *P* = 0.001, Figure 2d). Preterm infants showed increased heterogeneity (two‐sided Wilcoxon rank‐sum test, *P* < 0.001; median = 0.81 vs 0.76) of the gut resistome compared to term infants (Figure 2e), potentially suggesting complex external exposures of preterm infants, e.g., antibiotics or NICU environment. Comparable observations were further confirmed at the aARGs level using the same analyses as those for ARG subtypes (Figure S6a–e, Supporting Information). For example, six drug classes (e.g., quinolone, phenicol, and fosfomycin) from preterm infants showed higher abundance, and three drug classes (tetracycline, macrolide, and tigecycline) showed lower abundance than in term infants (linear mixed‐effect model, FDR < 0.05; Figure S6d, Supporting Information).

When predicting the potential host of aARGs based on their corresponding ORFs (*n* = 15914) from term infants, 28.0% ARG ORFs from 269 aARGs were assembled into MAGs and potentially distributed into 10 phyla, 53 families, 139 genera, and 326 species of bacteria (Figures S5d and S6f, Supporting Information). Compared with the top 10 known genera from term or preterm infants that respectively harbored 69.7% and 84.2% of total ARG ORFs with potential taxonomic assignment, ARG ORFs in term infants were mainly from *Bacteroides* (*n* = 812 ARG ORFs), *Klebsiella* (*n* = 627), *Phocaeicola* (*n* = 379), *Streptococcus* (*n* = 296), and *Bifidobacterium* (*n* = 214) (Figure 2f). In preterm infants, *Staphylococcus* (*n* = 10057) and *Klebsiella* (*n* = 7873) harbored the highest number of ARG ORFs. Differing from preterm infants, *Bifidobacterium* and *Bacterioides* were suggested as a potential source of ARGs in term infants, including *Bifidobacterium longum, Bifidobacterium breve*, *Bifidobacterium pseudocatenulatum*, *Bifidobacterium bifidum*, *Bacteroides uniformis*, *Bacteroides fragilis*, *Bacteroides ovatus*, *Bacteroides thetaiotaomicron*, and *Bacteroides xylanisolvens* (Figure 2f; Figure S5e, Supporting Information).

The proportion of plasmid‐borne ARG ORFs from term infants was lower than that of preterm infants (12.0% vs 27.2%; Chi‐squared test, *P* < 0.001). Among the potential bacterial hosts of plasmid‐borne aARGs, 94 clusters from 336 ORFs covering 71 aARGs were obtained, including 34 multi‐species clusters exclusively containing ARG ORFs that were taxonomically assigned, belonging to 53 known species. Unlike preterm infants, no other species dominated the distribution of plasmid‐borne ARGs, except *E. coli*, which was involved in 12 clusters, followed by *B. longum* (*n* = 8 clusters), *K. oxytoca* (*n* = 7 clusters), and *B. uniformis* (*n* = 6 clusters). At the gene level, we found that cluster 36 encoding *blaTEM/blaTEM‐1/blaTEM‐30* and cluster 41 encoding *aph(6)‐Id* were the most diverse groups with 11 species, respectively, followed by cluster 43 encoding *sul2* with nine species, and cluster 38 encoding *sul1*, with eight species (Figure 2g; Table S5, Supporting Information).

Collectively, these results revealed differences in the gut resistome between preterm and term infants in terms of not only their total burden and composition, but also the potential bacterial host and location of ARGs in the gut microbiome.

### 2.4 Differential Carriage of ARG Potentials from Bacterial Species in Preterm Infants

It has been suggested that the gut microbiome of preterm infants differs from that of term infants in both the composition and abundance of taxa,^[^ ^31^ ^]^ which was confirmed by our analysis, illustrated by the significantly different Bray–Curtis diversities at the bacterial species level (PERMANOVA, *P* = 0.001; Figure S7a, Supporting Information). However, variations in genome functions between the same species inhabiting the gut of preterm or term infants remain to be determined; thus, we sought to investigate changes in drug classes to which ARGs were resistant based on the bacterial species that were present in both preterm and term infants.

**Figure 3:**
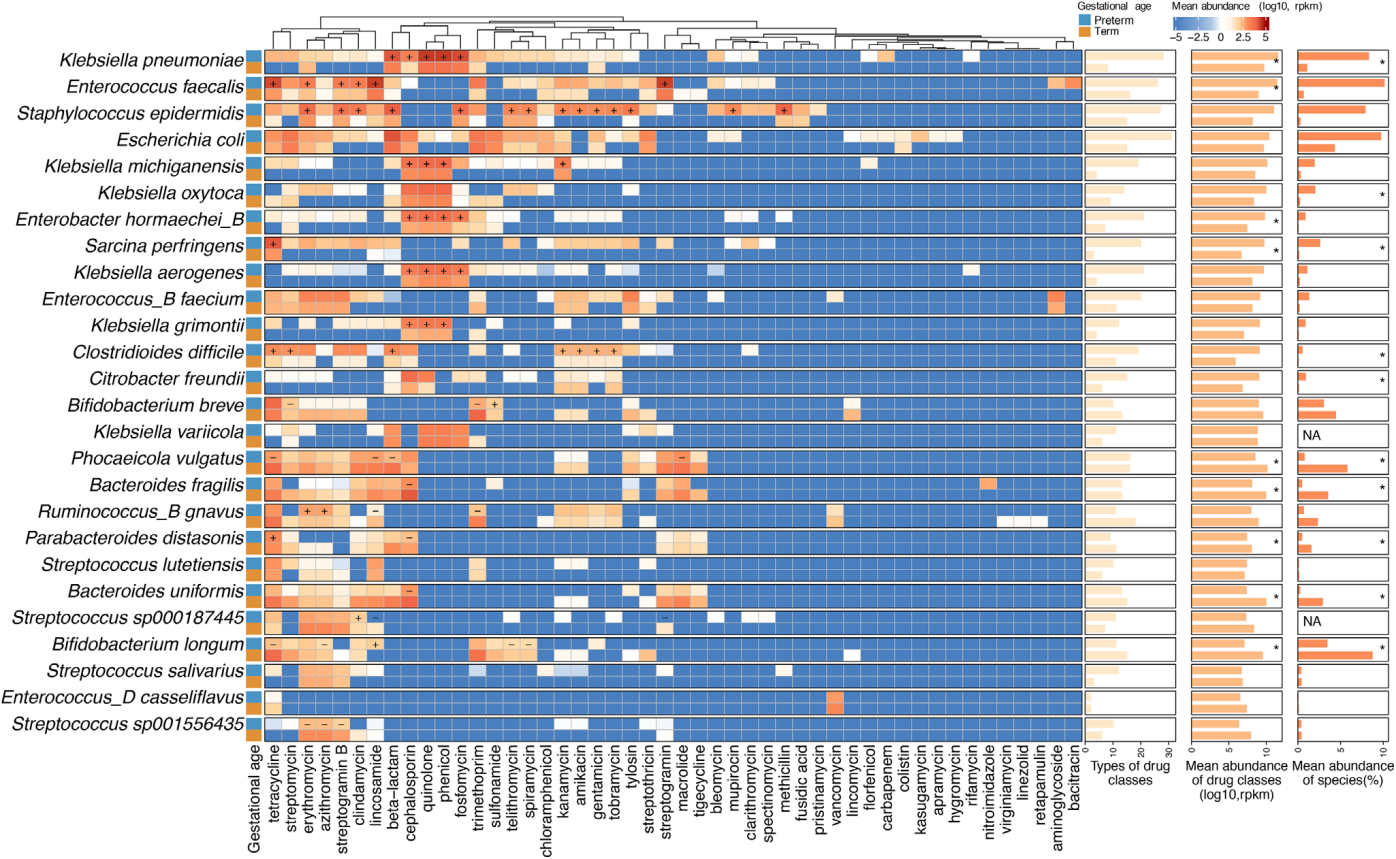
Differences in type and abundance of ARG drug classes from bacterial species present in both preterm and term infants. Only the bacterial species with > 0.5% prevalence in samples from infants born either preterm or term were considered, resulting in 26 species for this plot. The sign (+) and (–) indicate higher or lower (linear mixed‐effect model, *P* < 0.05) abundance of ARG drug classes of the corresponding column in the species of the corresponding row. The first and second bar plots from the right side indicate the mean abundance of bacterial species and drug classes, respectively, in preterm (the top bar in each row) and term (the bottom bar in each row) infants. The asterisk indicates a significant difference (linear mixed‐effect model, *P* < 0.05) between preterm and term infants in terms of the abundance of bacterial species or drug classes. The third bar plots indicate the total types of drug classes from each species in preterm or term infants. The color gradient indicates the mean abundance of ARG drug classes after log_10_ transforming. NA, the species were not detected by MetaPhlAn.

Among the potential bacterial species with ARGs, we focused on those with > 0.5% prevalence in both preterm and term infants in order to improve the confidence of the analysis. As a result, 26 potential species from 16 genera, including six from *Klebsiella*, four from *Streptococcus*, three from *Enterococcus*, and two each from *Bacteroides* and *Bifidobacterium*, were selected. We integrated the abundance of ARG drug classes within the same species on a per‐sample basis. Variations in the type, frequency, and abundance of ARG drug classes were detected in the same species in preterm or term infants (**Figure** **3**). Among the 26 species, four species harbored a significantly higher (linear mixed‐effect model, *P* < 0.05) abundance of ARG drug classes when comparing preterm infants to term infants, including *K. pneumoniae*, *E. faecalis*, *Enterobacter hormaechei_B*, and *Sarcina perfringens*; five species were less abundant with drug classes in preterm infants, including *B. longum*, *B. fragilis, B. uniformis*, and *P. vulgatus* (the second bar plot from the right side of Figure 3). Additionally, 25 drug classes involving 18 species were differentially present (linear mixed‐effect model, *P* < 0.05) in abundance between preterm and term infants. The drug classes of cephalosporin, phenicol, quinolone, fosfomycin, and tetracycline were more (linear mixed‐effect model, *P* < 0.05) abundant in ≥ 4 species from preterm infants; whereas the drug classes of lincosamide, cephalosporin, azithromycin, tetracycline, and trimethoprim were more (linear mixed‐effect model, *P* < 0.05) abundant in ≥ 2 species from term infants. Notably, *Bifidobacterium spp*. from term infants harbored more types and a higher abundance (linear mixed‐effect model, *P* < 0.05) of ARG drug classes, including trimethoprim, tetracycline, azithromycin, telithromycin, spiramycin, and streptomycin (the main plot of Figure 3).

This finding prompted us to investigate whether changes in the potential for resistance of each bacterial species were related to the different abundance in the gut microbiome of preterm or term infants. Thus, we characterized the bacterial composition of all stool samples from preterm and term infants using MetaPhlAn 4.^[^ ^32^ ^]^ Subsequently, we used Procrustes analysis to estimate the correlation between the gut bacteriome (species level) and resistome (aARGs), and observed a significant correlation between the composition of aARGs and the bacterial community as a whole from infants born preterm or term (M^2^ = 0.78 for preterm infants and 0.71 for term infants, *P* = 0.0001 for both), suggesting that the gut resistome of infants resembled their bacterial hosts. At the per‐species level, we found that species that were enriched in ARG drug classes in either preterm or term infants had a correspondingly higher (linear mixed‐effect model, *P* < 0.05) relative abundance in the bacterial community in infants (the first bar plot from the right side in Figure 3). Significant correlations (Pearson's r = 0.959, *P* < 0.001 for preterm infants; Pearson's r = 0.965, *P* < 0.001 for term infants) were also observed between mean abundances of ARG drug classes and species (Figure S7b, Supporting Information). These results suggested the bacterial community plays a significant role in the composition of the gut resistome in infants.

### 2.5 Dynamics of the Gut Resistome in Preterm Infants and Its Main Determinants

**Figure 4:**
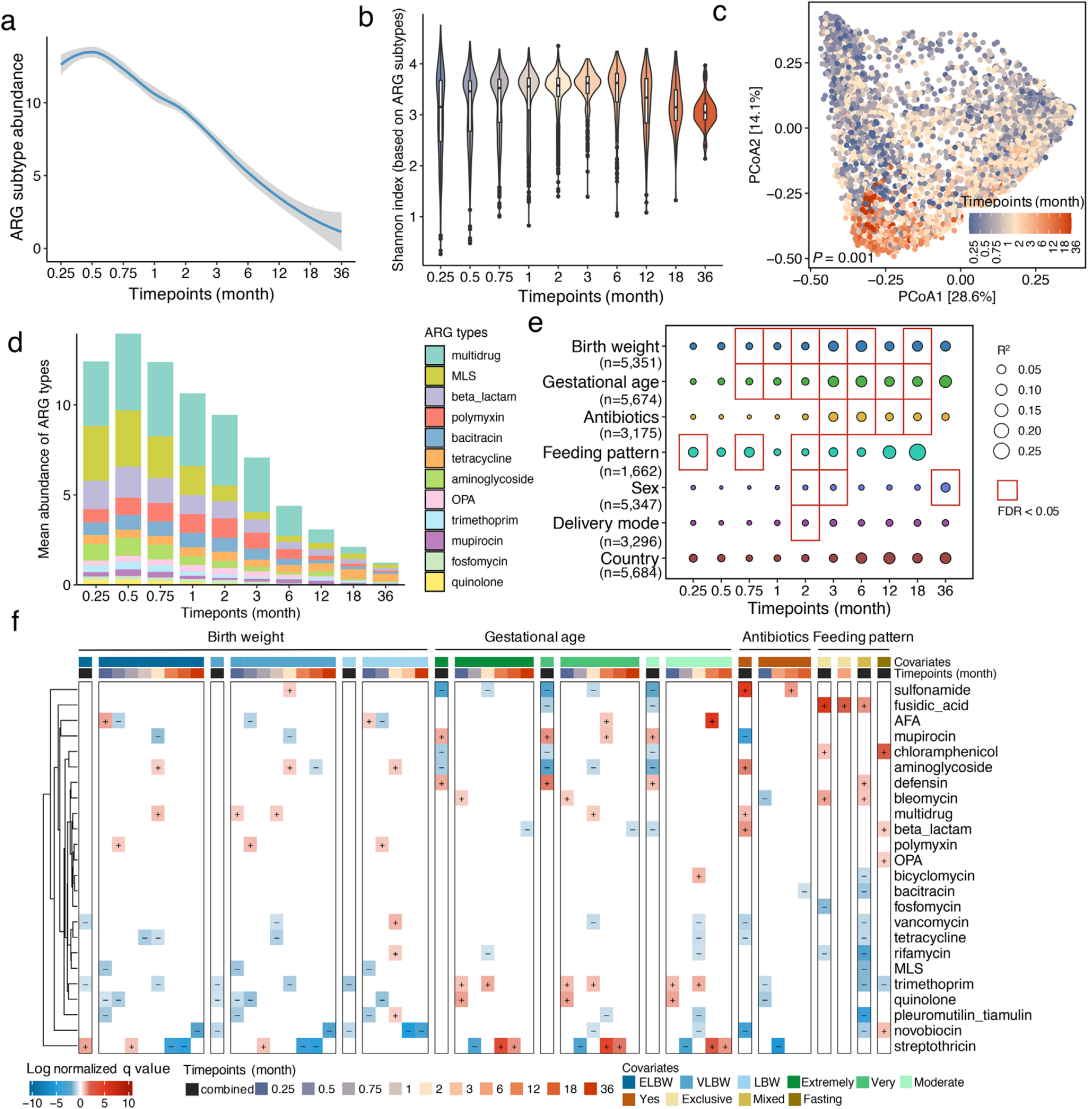
Dynamic composition and development of gut resistome in preterm infants. a, b) The total resistance load (a) and alpha diversity (i.e., Shannon diversity of ARG subtypes; b) in preterm infants decreased as the infant aged. ^*^FDR < 0.05 (two‐sided Wilcoxon rank‐sum test), NS: non‐significant. c) Principal coordinate analysis (PCoA) ordination of beta diversity of gut resistome measured by Bray–Curtis distances with ARG subtype abundance profiles (n = 5683 samples; PERMANOVA, *P* = 0.001). d) Changes of the abundance of ARG types in preterm infants. Only types with a prevalence > 10% and mean abundance > 0.1 capc are plotted. e) Significance and explained variance of seven clinical covariates at each of the timepoints, determined by PERMANOVA on between‐sample Bray–Curtis distances of ARG subtypes. f) Significant associations between specific ARG types and four clinical covariates (MaAsLin2; *q* < 0.25). The reference for each covariate as below: normal birth weight (≥ 2500 g, NBW) as reference birth weight with other levels of born extremely low birth weight (< 1000 g, ELBW), very low birth weight (1000–1500 g, VLBW), low birth weight (1500–2500 g, LBW); late preterm birth (34–37 weeks) as reference for gestational age with other levels of extremely preterm birth (< 28 weeks), very preterm birth (28–32 weeks), moderate preterm birth (32–34 weeks); free antibiotic exposure history before sampling as reference for infants with antibiotic exposure history before sampling; non‐breastfeeding as reference with other levels of exclusive breastfeeding, mixture breastfeeding with formula, and fasting when sampling. The sign (+) and (–) indicate positive and negative associations, respectively. The color gradient indicates the strength of the association calculated by *–log_10_(q‐value) × sign(coeff)*.

Longitudinal sampling enabled further investigation of the temporal dynamics of the gut resistome in preterm infants. We stratified the stool samples into ten discrete time points according to infant age, which were determined to maximize the number of samples within each window and reflect the succession of the gut microbiome. We found that the total burden of the gut resistome was relatively high after delivery and declined gradually over the first three years of life (i.e., having a median of > 10 capc within the first month and then declining to < 4 capc after the first year of life) (**Figure** **4**a). The Shannon diversity of ARG subtypes also gradually declined (two‐sided Wilcoxon rank‐sum test, FDR < 0.05; Figure 4b). Focusing on 12 ARG types that were present in > 10% samples and had a mean abundance > 0.1 capc, they accounted for > 95% of total ARG burden in all timepoints. Among them, six types, including MLS, beta‐lactam, aminoglycoside, trimethoprim, mupirocin, and quinolone, accounted for the majority of decrease in abundance during the first year of life; while other types of multidrug, polymyxin, bacitracin, OPA, and fosfomycin were stabilized with high abundance in the first three months of life and then decreased (Figure 4d; Figure S8, Supporting Information). The abundance of tetracycline increased further starting from the first six months of life. A similar pattern of tetracycline was confirmed in ARG drug classes using the assembly‐based approach (Figure S9a, Supporting Information).

Although there was marked heterogeneity in gut resistome of stool samples from preterm infants, the principal coordinate analysis ordination of Bray–Curtis distances of ARG subtypes still showed a strong longitudinal gradient as the infants grew (PERMANOVA, *P* = 0.001; Figure 4c for ARG subtypes; Figure S9b for aARGs, Supporting Information), together with other clinical covariates accounting for variances, including infant age at sampling, gestational age, antibiotic exposure, and feeding pattern (PERMANOVA, FDR < 0.01; Figure S9c for ARG subtypes, Figure S10a for aARGs, Supporting Information). More specifically, when stratifying samples into discrete timepoints, birth weight, gestational age, antibiotic exposure history before sampling, feeding pattern at sampling, sex, and delivery mode were significantly (PERMANOVA, FDR < 0.05) associated with the preterm infant gut resistome at one or more timepoints based on ARG subtypes (Figure 4e). Birth weight and gestational age, which are closely related (Pearson's r = 0.87, *P* < 0.001; Figure S10b, Supporting Information), predominantly contributed to the variations and exerted a long‐lasting effect on the development of the gut resistome. Surprisingly, the country was not detected to have a significant influence at either of the timepoints on the gut resistome in preterm infants. These observations based on ARG subtypes were highly similar to those based on aARGs (Figure S9d, Supporting Information). When examining the specific ARG types associated with clinical covariates, we found that variations in the associated ARG types depended on the covariates. For example, ARG‐type multidrug was positively associated with ELBW, VLBW, and antibiotic use; whereas quinolone was mainly negatively associated with birth weight, gestational age, and antibiotic use. Levels of novobiocin, PT, trimethoprim, MLS, rifamycin, tetracycline, vancomycin, fosfomycin, bacitracin, and bicyclomycin were negatively associated with exclusive or partial breastfeeding (Figure 4f).

### 2.6 Accelerated Convergence of Gut Resistome Preceding NEC Onset

The emergence of specific bacterial and viral features, including taxonomic composition and functional capacity, has been reported to precede NEC onset,^[^ ^4^, ^22^, ^33^ ^]^ which could facilitate the early detection of NEC and serve as potential clinical targets for therapeutics. However, no studies have examined how the gut resistome is altered in infants with NEC. To address this, we analyzed all publicly available stool samples with information on infant disease, that is, the clinical status of NEC. In total, 4180 stool samples collected from birth to 1331 days of life by seven public studies and our newly sequenced cohort were analyzed, covering 98 preterm infants who developed NEC (cases, *n* = 1168 samples) and 285 preterm infants free of NEC (controls, *n* = 3012 samples) that were matched for gestational age (two‐sided Wilcoxon rank‐sum test, *P* < 0.05), delivery mode, feeding pattern, and antibiotic exposure history before sampling (Chi‐squared test with “study” as a block factor, *P* < 0.05; Table S10, Supporting Information). When comparing all samples from cases (stratified into pre‐NEC and post‐NEC, the latter included samples collected on the day of NEC onset) and controls in the meta‐cohorts by calculating the Bray–Curtis distances of ARG subtypes and aARGs, samples from controls and pre‐NEC infants overlapped substantially, whereas PERMANOVA statistics were significant for ARG subtypes (*P* = 0.001, Figure S11a, Supporting Information) and aARGs (*P* = 0.001, Figure S11b, Supporting Information). The Shannon diversity of ARG subtypes and richness of aARGs were significantly increased (linear mixed‐effect model, *P* = 0.0006 for ARG subtypes, *P* = 0.016 for aARGs) in cases after NEC onset compared with pre‐NEC infants. No significant differences in the richness or Shannon diversity of gut resistome between controls and pre‐NEC infants (linear mixed‐effect model, *P* > 0.05) were observed for either approach. We further applied the above analyses to the bacterial profile of these stool samples, and samples from cases did not cluster separately from controls but showed a significant difference (PERMANOVA, *P* = 0.001; Figure S11c, Supporting Information). The richness and Shannon diversity of the gut bacteriome from pre‐NEC infants were significantly decreased (linear mixed‐effect model, *P* < 0.001 for richness and *P* < 0.001 for Shannon diversity; Figure S11c, Supporting Information).

**Figure 5:**
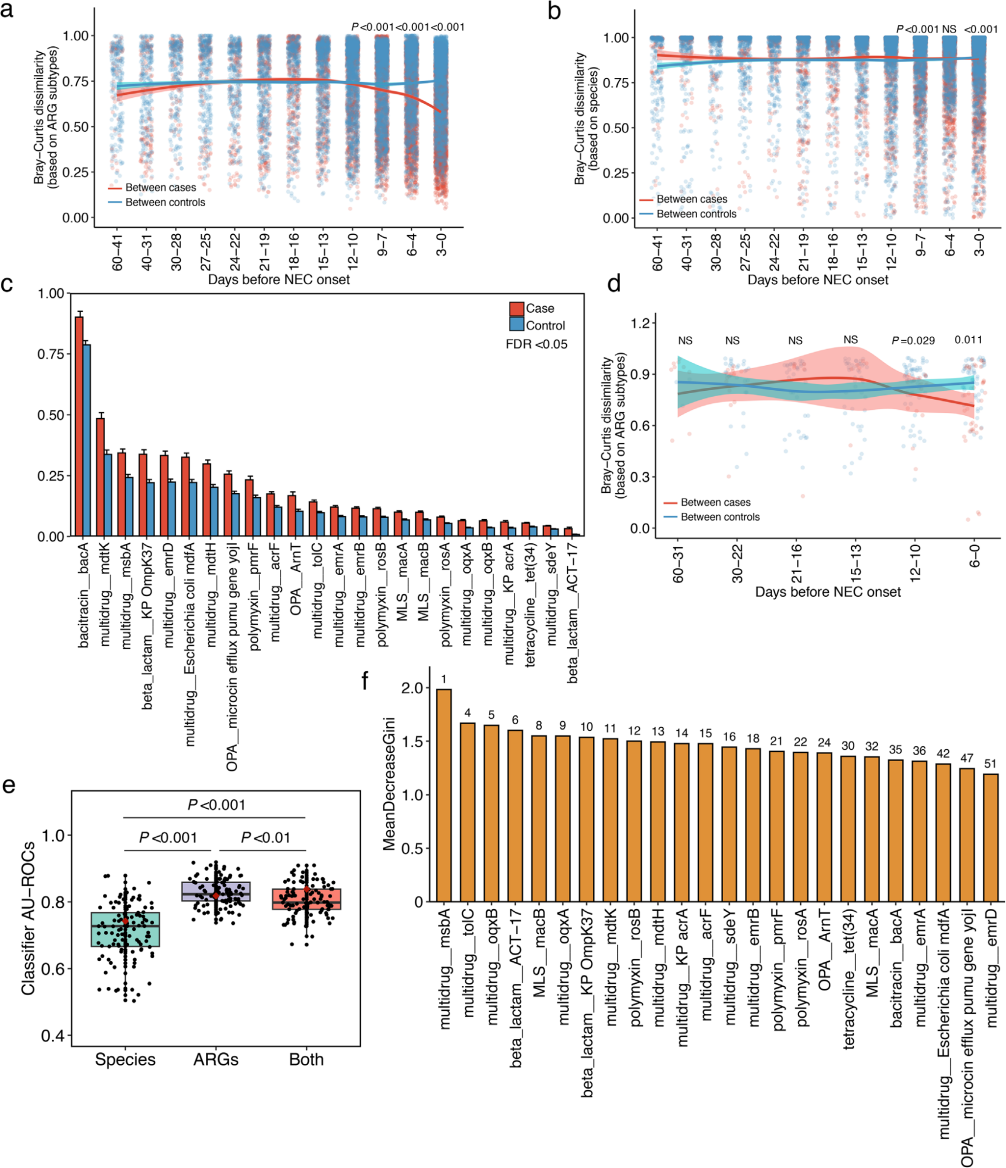
Accelerated convergence of gut resistome preceding NEC onset in preterm infants. a, b) Bray–Curtis distances between cases (red) and between controls (blue) based on ARG subtypes (a) and bacterial species (b) in sliding windows before NEC onset. The curves show locally estimated scatterplot smoothing (LOESS) fits for distances with 95% confidence intervals for each fit, as implemented in the geom_smooth function in the ggplot2 v3.5.1. Statistical significance at each window was assessed by a two‐sided Wilcoxon rank‐sum test. c) ARG subtypes with significant differences (linear mixed‐effect model, FDR < 0.05) between cases and controls from the convergent period, i.e., the first three sliding windows. All the values in the bar plots are presented as mean ± SE. d) Bray–Curtis distances between cases (red) and between controls (blue) based on ARG subtypes in sliding windows before NEC onset in an external validation preterm birth cohort. The curves show locally estimated scatterplot smoothing (LOESS) fits for distances with 95% confidence intervals for each fit, as implemented in the geom_smooth function in the ggplot2 R package. Statistical significance at each window was assessed by a two‐sided Wilcoxon rank‐sum test. e) Random forest with bootstrapping on the features to predict the onset of NEC in preterm infants with a model trained based on six public studies based on either ARG subtypes, bacterial species, or a combination of ARG subtypes and bacterial species. The red diamond indicates the AU‐ROC values from models without bootstrapping. f) Performance (assessed by the mean decrease in GINI coefficient) of predictive features of 24 NEC‐associated subtypes in the external validation preterm birth cohort. The number on the top of each bar indicates the rank in the total set of features.

To explore whether the dynamics of the gut resistome associated with NEC progression, we next focused on stool samples from cases before the onset of NEC (referred to as “pre‐NEC”), and matched samples from controls in the same cohort with comparable gestational age (± 0.5 week) and day of life (± 1 day) at sampling (Table S11, Supporting Information). We then used a sliding window, counting backward from the day of NEC onset, to examine the dissimilarity in the gut resistome between samples from cases and controls. Intriguingly, the dissimilarity of ARG subtypes between pre‐NEC samples decreased in sliding windows spanning the first three windows before NEC onset (i.e., nine days before NEC onset), which was significantly lower (two‐sided Wilcoxon rank‐sum test, *P* < 0.001) than that of controls within the same sliding window (**Figure** **5**a). This suggested that the beta diversity of the gut resistome in infants began to converge ≈9 days before NEC onset. Moreover, there was no observable convergence of the gut bacteriome in cases (Figure 5b), suggesting that the gut resistome convergence was not solely attributed to changes in the gut bacteriome before NEC onset.

Next, we explored the differentially abundant ARG subtypes during the convergence of the gut resistome in cases compared to controls (i.e., the first three sliding windows; *n* = 199 samples from cases, *n* = 367 samples from controls). The results showed that 24 ARG subtypes changed significantly in abundance (linear mixed‐effect model, FDR < 0.05), which were higher in cases than in controls (Figure 5c). Next, we tested these 24 differentially abundant ARG subtypes in each of the other sliding windows or together. Notably, no ARG subtypes showed significant differences between cases and controls > 9 days before NEC onset (linear mixed‐effect model, FDR > 0.05). These ARG subtypes enriched in cases mainly conferred antibiotic resistance to multidrug (*n* = 13 subtypes), polymyxin (*n* = 3), MLS (*n* = 2), OPA (*n* = 2), and beta‐lactam (*n* = 2), as well as bacitracin and tetracycline (*n* = 1 each) (Figure 5c).

### 2.7 External Validation and Predicting NEC Onset in an External Prospective Preterm Birth Cohort

To further validate the discovery of the convergent pattern of ARGs in preterm infants before NEC onset, we analyzed an external preterm birth cohort who were longitudinally sampled multiple times until NEC onset (Table S10, Supporting Information). We applied the same analyses in the public cohorts to match the samples from cases and controls, which resulted in 34 pre‐NEC samples from nine cases and 37 matched samples from 16 controls. Due to the limited number of samples that could not cover all sliding windows in the discovery meta‐cohorts, we merged parts of the sliding windows to increase the analysis power.

Using the same analyses, we confirmed that the distance of ARG subtypes between pre‐NEC samples was significantly lower (two‐sided Wilcoxon rank‐sum test, *P* < 0.05) than that of controls six days before NEC onset (Figure 5d), even though with a lack of pre‐NEC samples from window 7–9 days. This did suggest that the gut resistome in preterm infants before NEC onset converged.

Given the clinical importance of predicting NEC onset, we further tested whether the gut resistome during the convergent period could be used as a tool to prescreen infants before NEC onset. The gut resistome predictive model trained with public cohorts showed higher (*P* < 0.001; two‐sided Wilcoxon rank‐sum test) accuracy of 0.823 as quantified by the area under the receiver operating characteristic curve (AU‐ROC) (median; 95% CI, 0.819–0.834; Figure 5e) in the external validation cohort, which was higher than the predictive performance of the model with bacterial species (AU‐ROC = 0.727, 95% CI, 0.696–0.728). Predicting with a combination of the ARG subtypes and bacterial species did not improve the performance further (AU‐ROC = 0.798, 95% CI, 0.797–0.815) (Figure 5e), highlighting the importance of the gut resistome in models. We then investigated the importance of 24 NEC‐associated ARG subtypes in differentiating cases and controls based on the mean decrease in the GINI coefficient (i.e., MeanDecreaseGINI). The results showed that these 24 NEC‐associated ARG subtypes contributed 14.3% of the total MeanDecreaseGINI in the model, and 7 and 23 were among the top 10 and 50 most important features, respectively (Figure 5f). Three subtypes of multidrug__*mdtK*, multidrug__*msbA*, and MLS_*macB* were the most predictive ARG subtype in the model. The other subtypes in the top 10 included beta‐lactam_*ACT‐17*, beta_lactam__*K. pneumoniae OmpK37*, polymyxin__*pmrF*, and multidrug__*acrF* (Figure 5f; Table S12, Supporting Information).

## 3 Discussion

Concurrent with the overuse of antibiotics and extended hospitalization, the prevalence of gut resistome in preterm infants has become a public health concern. Nevertheless, there is a gap in the literature with respect to the generalizable composition and development of the gut resistome in preterm infants across multiple cohorts. With the increasing number of birth cohorts and the availability of metagenomic datasets, cross‐cohort analysis has emerged as an effective method to identify reproducible features (e.g., microbiome transmission, microbial genome catalogues)^[^ ^34^, ^35^ ^]^ or associations/biomarkers of microbiome with various diseases, despite variations in individual cohort characteristics.^[^ ^36^, ^37^ ^]^ Here, we conducted large‐scale, cross‐cohort, read‐ and assembly‐based analyses to discover the landscape of the gut resistome in preterm infants, including the predominant components of ARGs, their associated hosts, shaping factors, and the potential for prediction of NEC onset in early life.

Regardless of the types and courses of antibiotics that were administered to preterm infants that were not available in the majority of included studies, we observed up to 15 (median) ARG types from the read‐based approach and 13 (median) ARG drug classes from the assembly‐based approach in the gut resistome. The main agents present in high abundance and prevalence in preterm infants were multidrug, MLS, beta‐lactam, polymyxin, and tetracycline, which have been classified by the WHO as important, highly important, or critically important.^[^ ^38^ ^]^ We propose that the high diversity of ARGs in the gut microbiome of preterm infants likely stems from multiple factors beyond antibiotic exposure alone, including prolonged hospitalization^[^ ^13^ ^]^ and diverse environmental exposures (e.g., living in rural or urban areas).^[^ ^15^ ^]^ For example, ARGs for tetracycline, which is rarely prescribed during infancy (we indeed found no such prescriptions in infants for newly sequenced samples), were highly present and prevalent in preterm infants, and have been proposed to be obtained by infants vertically or horizontally.^[^ ^39^ ^]^ In addition, the meta‐cohort analyses enabled us to identify cohort‐specific ARG types and drug classes, illustrating that the gut resistome in infants varied across hospitals and countries, which was consistent with findings from a recent meta‐analysis on the early‐life gut resistome.^[^ ^40^ ^]^ From a clinical standpoint, these findings emphasize the necessity of local profiling of the gut resistome to effectively manage antibiotic stewardship and infections in the NICU using accurately targeted antimicrobial therapies instead of straightforward broad‐spectrum antibiotics.

It has been evidenced that ARGs are mobile elements and are likely to be hosted by multiple commensal species or opportunistic pathogens.^[^ ^40^ ^]^ However, the investigation of potential ARG hosts within the gut microbiome of preterm infants is still in its nascent stage. We found that ARGs in preterm infants were mainly carried by potential *Staphylococcus* and *Klebsiella*, whereas ARGs in term infants were potentially carried by *Bacteroides*, *Phocaeicola*, and *Bifidobacterium* as well as *Klebsiella*. At the functional level, previous studies have indicated that early‐life antibiotic consumption markedly disrupts the overall functional capacity of the gut microbiome, such as short‐chain fatty acid synthesis; however, changes in the genomic functions of individual bacterial species have not been investigated. Here, in conjunction with the observed differences of the gut resistome in preterm infants, we found that individual bacterial species concurrently inhabiting both preterm and term infants harbored differential potential of resistance to drug classes; that is, the opportunistic pathogens from preterm infants were more drug‐resistant whereas the beneficial species from term infants had higher resistant potential to drugs, reflecting ARG carriage as a strain‐specific trait and a clear strain shift of genomic functions in preterm and term infants. Evolutionarily, these notable changes corroborate the notion that specific molecular functions of the gut microbiome are selectively adapted to extrinsic pressures, which has been evidenced in the case of breastfeeding that could enrich HMO processing and utilization genes in the genome of *B. longum* strains.^[^ ^41^ ^]^ Additionally, Xu et al. reported that the development of gut resistome early in life was shaped by changes in the microbial capacity of carbohydrate metabolism.^[^ ^42^ ^]^ Overall, our results highlight the critical role of the ecological niche conferred by gestational age at birth in affecting the carriage of AMR in the gut bacteriome;^[^ ^40^ ^]^ however, the detailed molecular mechanisms that drive shifts in the genomic evolution of ARG require further investigation.^[^ ^42^ ^]^

Antibiotic therapy, one of the key medical advances in the last century, substantially reduces global infection‐related morbidity and mortality; however, one of the unintended consequences of antibiotic use worldwide is the rising number of bacteria with AMR.^[^ ^7^ ^]^ Although massive efforts have been made to mitigate the increase and spread of AMR, completely eradicating ARGs from the gut microbiome appears to be implausible, which underscores both the challenge and opportunity of leveraging the gut resistome to effectively predict or manage the progression of diseases. Indeed, Shuai et al.^[^ ^43^ ^]^ reported that the profile of the gut resistome in adults is associated with the progression of diabetes; in another study, it was found that some specific ARGs could serve as potential biomarkers for predicting autism spectrum disorder in children.^[^ ^44^ ^]^ In the present study, we focused on the dynamics of the gut resistome in preterm infants with NEC, which is a common and serious complication of preterm birth with an unclear pathogenesis. We analyzed public cohorts that provided detailed information on NEC onset and observed a convergence of the gut resistome with specific ARGs in the days before NEC onset. We further validated this trend in an external preterm birth cohort. Intriguingly, the gut bacteriome, a carrier of the gut resistome, did not converge in preterm infants in the same periods before NEC ensued. Although the profile of the gut resistome has been thought to be intimately connected with the composition of the gut microbiome, our results revealed that in an unhealthy state, this connection was not absolute, and changes in the gut resistome could not be fully explained by shifts in bacterial composition. The reason for the discrepancies in the changes in bacteria and their intrinsic genomic components remains to be investigated. Robust evolutionary adaptations in the phenotype or genomic functions of bacteria to extrinsic selection pressures (e.g., inflammatory responses, nutrient deficiency, antibiotic use, and hypoxia) occur frequently, enriching and maintaining ARGs that confer a growth advantage to the host.^[^ ^10^, ^45^ ^]^ In addition, viruses are known to contribute to the acquisition, preservation, and dissemination of ARGs,^[^ ^46^ ^]^ and given that a convergence of viral communities 10 days before NEC onset has been identified,^[^ ^22^ ^]^ the possibility of viral‐driven changes in the gut resistome before NEC onset is worthy of further investigation. Most notably, the specific ARGs that were differentially abundant between cases and controls in the converged period did not show any significant differences in other time windows, highlighting them as potential NEC‐associated ARGs. The classifier trained with ARG features via a machine learning algorithm could potentially distinguish preterm infants as healthy or at high risk of developing NEC for the external validation preterm birth cohort, outperforming the previously predictive model with the bacteria.^[^ ^4^ ^]^ Although the causative relationship between changes in the gut resistome and NEC onset could not be determined, these results highlight ARGs as potential targets for biomarker development to stratify the pre‐NEC state in preterm infants, but further investigations from multi‐center studies with larger sample sizes are necessary to refine the predictive model. Importantly, we anticipate that monitoring the trend or specificity of ARGs in the gut of preterm infants holds potential as a reliable proxy to accurately support the clinical prediction of NEC and treatments (e.g., guiding the choice of antibiotic administration).

Given the inherent difficulties in collecting longitudinal samples from preterm infants prior to NEC onset in clinical practice, the number of samples is likely to be small. Here, we utilized the first and largest metagenomic dataset from infants with NEC to date, which allowed us to identify associations between the gut resistome and NEC progression by integrating new and public samples. There are still several limitations and improvements that we should take into consideration for future research. First, the still rather limited sample size of case infants in the meta‐cohorts (*n* = 98) and validation cohort (*n* = 9) is certainly a limitation. Due to the low incidence of NEC in preterm infants (i.e., ranging from 1% to 12%),^[^ ^17^ ^]^ multi‐center collaborative research with a large sample size of case infants and low technical heterogeneity is desired to substantiate our findings. Additionally, the NEC‐associated ARG features were in silico identified and not validated with experimental techniques (such as quantitative PCR) due to inadequate amounts of DNA after sequencing, a typical challenge for sampling from infants, and which is even more problematic in those born preterm. With explicit experimental designs, the mechanisms driving the convergence of the gut resistome before NEC onset should be elucidated, with detailed clinical variables, such as antibiotic type and duration, or animal models for molecular exploration. Notably, dynamic changes in the gut metabolomics or proteomics prior to NEC onset in preterm infants are still missing, but warrant consideration in the future, given that certain gut tricarboxylic acid metabolites from a single time point exhibit potential to predict NEC onset.^[^ ^18^ ^]^ Third, all metagenomic sequencing data were based on paired‐end short‐read shotgun sequencing, which inherits some limitations from the short length of the initial sequencing reads and fragmented assembled contigs. Future studies with a long‐read sequencing technique, a hybrid approach of short‐ and long‐read sequencings, or Hi‐C in conjunction with shotgun sequencing that improves the capacity of detecting ARG‐bacterial host associations by crosslinking genomic DNA before cell lysis^[^ ^10^, ^47^ ^]^ would further shed light on novel patterns or biomarkers associated with NEC progression, and the host of ARGs that are typically located on plasmids. All these efforts will significantly refine the current findings. Lastly, studies that effectively translate the metagenomic findings into clinical practice for the early diagnosis and prevention of disease occurrence are essential.

In conclusion, our integrative analyses across diverse populations of preterm infants provide a systematic overview and advance our understanding of the gut resistome in early life. The disclosed main types of ARGs and their targeted agents, in terms of their prevalence and abundance at the population level, will improve our scientific knowledge of this health threat in preterm infants. This knowledge will serve as a useful guide for public health interventions with the aim of mitigating the broader spread of AMR. The discovery of a convergent gut resistome preceding the onset of NEC potentially implies its potential to predict the health status of preterm infants. These insights suggest that further investigation to use the gut resistome as an NEC predictor in clinical practice would open novel avenues for clinical interventions to prevent life‐threatening diseases.

## 4 Experimental Section

### Public Metagenomic Sequencing Datasets

PubMed with terms “(preterm) AND (metagenomics)” was combined to search studies that included sequencing data from the stool of preterm infants (< 37 gestational weeks) from birth to three years old (up to March 2023). Samples were then manually curated to remove those that did not correctly match the metadata. All samples were further manually included from preterm infants that were used to build the catalogue of early‐life human gut microbial genomes.^[^ ^35^ ^]^ Additionally, two studies sampling a large number of preterm infants who developed NEC were published when this manuscript was in review, which were also included.^[^ ^48^, ^49^ ^]^ As a result, the present study finally included a total of 5684 stool samples exclusively from preterm infants from birth to three years old, comprising 5505 samples publicly available from 18 cohorts and also 179 samples newly sequenced in this study (detailed cohort description stated below), to explore the landscape of gut resistome in preterm infants. The metadata of infants, including gestational age, delivery mode, birth weight, sex, feeding pattern (exclusive breastfeeding, exclusive formula, mixture breastfeeding with formula, solid food, or fasting), and if antibiotics were historically exposed before sampling (yes or no), were curated manually with maximal efforts (Table S1, Supporting Information). No statistical methods were used to pre‐determine sample sizes, but the current study already represented the largest dataset of longitudinal stool metagenomes of preterm infants to investigate the gut resistome.

### Description of Newly Sequenced Metagenomic Dataset

A total of 61 preterm infants born at < 34 weeks’ gestation were recruited by West China Second University Hospital in 2022 from Sichuan province, China. The protocol of this study was approved by the Ethics Committee of West China Second University Hospital (2022‐067). Informed consent was obtained from the parents of the included infants. All infants were cared for in the NICU at West China Second University Hospital, and NEC was diagnosed based on Bell's modified criteria by combining clinical, radiological, and/or histopathological evidence. Stool samples were collected longitudinally at multiple timepoints until the discharge (i.e., within 72 h, 1 week, 2 weeks, 4 weeks, and the day before discharge if possible) using sterile collection tubes for stool. The stool samples were collected by the hospital staff from the diapers and frozen at −40 °C immediately upon collection, and then moved to a −80 °C facility within 72 h until further analysis. The genomic DNA was extracted from a stool sample using the Magnetic Soil and Stool DNA Kit (TIANGEN Biotech Co., Ltd., China), and the sequencing library was prepared with NEBNext Ultra DNA Library Prep Kit for Illumina (NEB, USA) following the manufacturer's protocol. The qualified libraries were proceeded to paired‐end (2 × 150 bp) metagenomic sequencing on the Illumina Novaseq 6000 platform (Novogene Co., Ltd., China) at a targeted depth of 8 Gb.

### Description of the External Preterm Birth Cohort for Validation

An additional set of stool samples from an independent preterm infants born at ≤ 29 weeks’ gestation that were recruited at three NICUs within the Guangdong province from 2021 to 2022, which was approved by the Ethics Committee of Nanfang Hospital of Southern Medical University (NFEC‐2021‐054) from China. Nine preterm infants who developed NEC and 16 preterm infants without NEC as controls were selected among this additional cohort and sampled prospectively and longitudinally with parental consent, resulting in 34 and 37 stool samples from NEC and control infants, respectively, which were frozen at −20 °C immediately upon collection, and then moved to a −80 °C facility within 48 h until further analysis. Genomic DNA was extracted from stool samples with the ALFA‐SEQ Advanced Soil DNA Kit, and shotgun metagenomic sequencing was performed by the Illumina Novaseq 6000 platform (Magigene Ltd., China) at a targeted depth of 6 Gb (2 × 150 bp paired‐end reads).

### Metagenomic Pre‐Processing and Taxonomic Profiling

The raw metagenomic reads newly sequenced in both the discovery and external validation cohorts were quality‐controlled by removing TruSeq3 adapter sequences and reads less than 50 nucleotides in length, and human reads (hg19 human reference genome from https://huttenhower.sph.harvard.edu/kneadData_databases/) were filtered with KneadData v0.10.0 (https://github.com/biobakery/kneaddata). The publicly available metagenomic reads were downloaded from the NCBI SRA based on the accession numbers of each included study, which were then subjected to quality‐control and human contamination filtering using KneadData v0.7.2 with default values before subsequent analyses. To minimize the influence of sequencing depth across samples on building the landscape of gut resistome, metagenomes with > 2 million quality‐controlled reads from discovery meta‐cohorts were used. The taxonomic profiling of quality‐controlled metagenome was determined using MetaPhlAn 4 v4.0.2^[^ ^32^ ^]^ with the default settings, and the unclassified fraction was estimated with the option “–unclassified_estimation”.

### Composition and Abundance of ARG Types and Subtypes in Samples from the Read‐Based Approach

The profile and abundance of ARG types and subtypes were identified directly from the quality‐controlled reads by using ARGs‐OAP v3.2.2^[^ ^28^ ^]^ with default parameters. The dependent database of structured ARGs (SARG) v3.0, as one of the most popular ARG databases, was robustly constructed based on CARD, the ARG database (ARDB), and the NCBI NR database, with a manual curation for accurate classification, reflecting the up‐to‐date number of non‐redundant ARG deposited sequences. Each ARG sequence in the SARG database is tagged with its functional gene annotation (ARG subtype) and the class of antibiotics to which the gene confers resistance (ARG type), totaling 30 ARG types, 2744 ARG subtypes from 12746 ARG sequences that could be correctly annotated using metagenomic short reads. Additionally, ARGs‐OAP includes the algorithm to estimate the cell number, which could be used for normalization of the ARG abundance, expressed as “copies of ARGs per prokaryote's cell (capc)” in each sample, as stated in the context. The *qac* genes in the output of ARGs‐OAP were filtered out.

### Metagenomic Assembly, Annotation, Abundance, and Taxonomic Assignment of ARGs

Quality‐controlled reads from each sample were assembled using MegaHIT v1.1.3^[^ ^50^ ^]^ with the option “–min‐contig‐len 1000”. The MAGs from each sample were generated using three metagenomic binning tools (MetaBAT v2.12.1,^[^ ^51^ ^]^ MaxBin v2.2.6,^[^ ^52^ ^]^ and CONCOCT v1.0.0^[^ ^53^ ^]^) using metaWRAP v1.3.1^[^ ^54^ ^]^ with default parameters. The shortest contig length for binning was set as the default with 1000 bp, except for MetaBAT, which requires at least 1500 bp. Afterward, MAGs within each sample were refined and dereplicated with the Bin_refinement module of metaWRAP with options ‘‐c 50 ‐x 10’, corresponding to the criterion of medium‐quality draft MAGs.^[^ ^55^ ^]^ To remove the chimeras, the dereplicated MAGs were further quality‐checked by GUNC v1.0.5.^[^ ^56^ ^]^ Taxonomic annotation of MAGs was conducted by GTDB‐Tk v2.4.0^[^ ^57^, ^58^ ^]^ (reference database version R220) with ‘classify_wf’ workflow using default parameters. The NCBI taxonomy annotation was provided using the “gtdb_to_ncbi_majority_vote.py” script available in the GTDB‐Tk repository, which was used to match the species profiled by MetaPhlAn.

The ARGs were annotated by aligning contigs against the NCBI's curated Reference Gene Database and curated collection of Hidden Markov Models using its accompanying resistance gene identifier (AMRFinderPlus v4.0.3) with default settings.^[^ ^59^ ^]^ To keep only ARGs against antibiotics, the genes against quaternary ammonium were filtered. The predicted DNA sequences of ARG ORFs from AMRFinderPlus outputs were extracted from each sample, and quality‐controlled reads were mapped back to these ORFs using BWA‐MEM^[^ ^60^ ^]^ implemented in CoverM v0.6.1 (https://github.com/wwood/CoverM). The abundance of each ORF in each sample was then estimated by “contig” mode of CoverM v0.6.1 with option ‘–min‐read‐percent‐identity 95, –min‐read‐aligned‐percent 50, –min‐covered‐fraction 75′ to keep high‐quality mappings, and the results were expressed as rpkm (reads per kilobase per million mapped reads). That is, the quality‐controlled reads were excluded if the percent identity and aligned bases were less than 50% and 95%, respectively, and the minimum coverage of each ORF for its presence was 75%. The rpkm has been commonly used for relative abundance comparisons with metagenomes, which normalizes the abundance based on gene length (in kilobases) and sequencing depth (per million reads).^[^ ^61^ ^]^ The abundance of ARG drug classes (Subclass information from AMRFinderPlus output) was summed from the abundance of ORFs belonging to that confer resistance to those drug classes in the given sample. The host of aARGs was determined by taxonomic assignments of MAG on which their ORFs were located.

### Plasmid‐Borne Sequences Identification and Cluster of ARGs

To predict the location of ARGs, we used the whole contigs where ARG ORFs were located for classification using Platon v1.7^[^ ^62^ ^]^ with the ‘–meta’ option, and PLASMe v1.1^[^ ^63^ ^]^ with default settings. Contigs were classified to be plasmid‐borne only when the two tools simultaneously reported it. The plasmid‐borne ARG ORFs with taxonomical assignments were further clustered using CD‐HIT‐EST v4.8.1^[^ ^64^ ^]^ based on their nucleotide identity of > 99% over 90% coverage of ORFs (option ‘‐c 0.99 ‐g 1 ‐aL 0.9 ‐aS 0.9′).

### Statistical Analysis—Procrustes Analysis

Correlations between the profile of aARGs and the bacterial species within each metagenome were analyzed using the Procrustes algorithm.^[^ ^65^ ^]^ The abundances of aARGs and bacterial species were Hellinger‐transformed, followed by Bray‐Curtis distances calculation. The symmetric correlation coefficient and *P* value were calculated with 9999 permutations using with ‘protest’ function.

### Effect Size Analysis

The proportion of explained variance (R^2^, effect size) and significance of clinical covariates in terms of ARG subtypes or aARGs were estimated based on all samples together or stratified by age into distinct timepoints (as described below), which were then quantified by PERMANOVA calculated from Bray‐Curtis distances. When calculating the R^2^ value of each covariate, samples without metadata available for the given covariate were filtered before running each PERMANOVA with 1000 permutations, taking “study” as a blocking factor by the “adonis2” function from the R package “vegan” v2.6‐4.^[^ ^66^ ^]^ The covariates included subjects, infant age, gestational age, birth weight, antibiotic exposure history before sampling (yes vs no), feeding pattern at sampling (exclusive breastfeeding, mixture breastfeeding with formula, non‐breastfeeding, and fasting), delivery mode (vaginal vs C‐section), gestational age (term vs preterm), sex (female vs male), and country.

### MaAsLin2 Analysis

Significant associations between ARG types and clinical covariates (including gestational age, birth weight, infant age, antibiotic intervention, delivery mode, feeding pattern at sampling, sex, and country) were examined with a multivariate linear mixed modelling as implemented in MaAsLin2 v1.10.0.^[^ ^67^ ^]^ All investigated clinical covariates were included as fixed effects, with “subjects” as a random effect to account for the potential heterogeneity across studies. A prevalence threshold of 5% was set for ARG type selection, and the normalized relative abundances with total sum scaling (TSS) were then arcsin‐square root‐transformed (AST) before analyses. The gestational age was grouped into extremely preterm birth (< 28 weeks), very preterm birth (28–32 weeks), moderate preterm birth (32–34 weeks), and late preterm birth (34–37 weeks), which was used as reference. The birth weight was grouped into extremely low birth weight (< 1000 g, ELBW), very low birth weight (1000–1500 g, VLBW), low birth weight (1500–2500 g, LBW), and normal birth weight (≥ 2500 g, NBW), which was used as reference. Infants without an antibiotic exposure history before sampling were used as a reference for infants with an antibiotic exposure history before sampling. The feeding pattern at sampling was grouped into exclusive breastfeeding, mixed breastfeeding with formula, fasting, and non‐breastfeeding, which was used as a reference.

### NEC and Matched Control Samples

The day of life (DOL) of NEC onset in cases was collected from the metadata of included studies, and days between each sampling and NEC onset were thus calculated. Then a sliding 3‐day window was used from the first month, counting backward from the day of NEC onset, followed by a 10‐day window and a 20‐day window in the second month due to limited samples. In each sliding window, only one sample from an infant with a closer time to NEC onset was chosen. The matched samples from controls recruited in the same cohort with comparable gestational age (± 0.5 week) and day of life (± 1 day) at sampling were included. If multiple samples from the same infant matched with the targeted cases, the sample collected at an older DOL was kept. Finally, the included stool samples of controls in each sliding window were dereplicated in cases where some samples matched more than once. Overall, only one sample from each infant was kept in each sliding window. Bray–Curtis distances of gut resistome based on the abundance matrix of ARG subtypes between cases or controls were calculated as a function of time preceding NEC onset.

### Random Forest‐Based Machine Learning Approach

The abundances of ARG subtypes and bacterial species were used for machine learning analysis to predict NEC onset in preterm infants. Only studies with the minimal number of samples in either positive or negative group (i.e., infants with or without NEC) from 9 days before NEC onset, with five were included. Therefore, six studies (BrooksB_2017, LouYC_2024, MasiAC_2021, RahmanSF_2018, RavehSadkaT_2016, ThanertR_2024) were included to train the predictive model using the function randomForest from the R package “randomForest” v4.7‐1.1^[^ ^68^ ^]^ with 500 estimator trees. The optimal number of “mtry” option in the model for the best predictive performance was tuned from 1 to the total number of features present in samples, depending on groups of features (i.e., 1633 ARG subtypes, 515 bacterial species, and 2148 ARG subtypes + bacterial species). Prediction and performance of the model were estimated with the “predict”, “prediction”, and “performance” functions from the R package “ROCR” v1.0‐11^[^ ^69^ ^]^ and “pROC” v1.18.4.^[^ ^70^ ^]^ To statistically analyze the significance between groups of features, the machine learning models were bootstrapped on each group of features (ARG subtypes, bacterial species, and ARG subtypes + bacterial species) 100 times, which produced 100 AU‐ROC values for each group of features. Theoretically, bootstrapping on the features provided an estimation of how dependent the model performance is on any one specific combination of features. The importance of predictive features was obtained from the model without bootstrapping based on values of MeanDecreaseGINI from the “importance” function of the R package “randomForest”.

### Stratification of Stool Samples

To quantify the dynamics of the gut resistome in preterm infants, continuous infant age was grouped into nine specific age timepoints, which were determined to both keep the maximal number of samples in each timepoint and also precisely reflect the gut resistome development. The nine timepoints included: month 0.25 (0–7 days; *n* = 413; median of days (MD) = 5, IQR = 3–7 days), month 0.5 (8–14 days; *n* = 989; MD = 11, IQR = 9–13 days), month 0.75 (15–21 days; *n* = 1052; MD = 18, IQR = 16–20 days), month 1 (22–30 days; *n* = 947; MD = 26, IQR = 23–28 days), month 2 (31–60 days; *n* = 1375; MD = 41, IQR = 36–49 days), month 3 (61–90 days; *n* = 313; MD = 70, IQR = 64–77 days), month 6 (91–180 days; *n* = 206; MD = 121, IQR = 100–139 days), month 12 (181–360 days; *n* = 197; MD = 237, IQR = 209–271 days), month 18 (361–540 days; *n* = 107; MD = 422, IQR = 371–484 days), and month 36 (> 541 days; *n* = 84; MD = 730, IQR = 614–922 days).

To account for the potential heterogeneity across studies and repeated measures, comparisons and significances of features (including the alpha diversity, bacterial, ARG and drug classes profiles) in relation to the gut resistome between groups (i.e., preterm and term infants or infants with or without NEC) were determined using a linear mixed‐effect model with “study” and “subjects” as random factors implemented in R package “lmerTest” v3.1‐3.^[^ ^71^ ^]^ Other specific statistical analyses were otherwise stated in the relevant content.

All other quantification and statistical methods have been provided in the relevant context, with *P* value or adjusted *P* value for multiple comparisons by Benjamini‐Hochberg FDR method^[^ ^72^ ^]^ (FDR value). A *P* value or FDR < 0.05 was considered statistically significant. In addition, the data in accordance with the “Strengthening The Organization and Reporting of Microbiome Studies” (STORMS) guidelines for the human microbiome research was reported (Table S13, Supporting Information).^[^ ^73^ ^]^

### Patient and Public Involvement

Patients and/or the public were not involved in the design, conduct, reporting, or dissemination plans of this research.

## Conflict of Interest

The authors declare no conflict of interest.

## Author Contributions

S.Z., H.W., L.Z., S.L., and Y.Y. contributed equally to this work. S.Z., H.W., D.M., and S.W. conceived the study. S.Z. and S.W. performed the analyses. S.Z., H.W., L.Z., S.L., Y.Y., M.T., Y.H., J.H., R.Z., Y.H., J.F., W.H., Y.L., W.S., and S.W. contributed to sample and data collection. J.Y., M.Z., F.Z., X.S., Q.L., Y.Q., and Z.Z. assisted in the interpretation of findings. S.Z. and S.W. drafted the manuscript. All authors read, edited, and approved the manuscript.

## Supporting information



Supporting Information

Supplemental Table 1

Supplemental Table 2

Supplemental Table 3

Supplemental Table 4

Supplemental Table 5

Supplemental Table 6

Supplemental Table 7

Supplemental Table 8

Supplemental Table 9

Supplemental Table 10

Supplemental Table 11

Supplemental Table 12

Supplemental Table 13

## Data Availability

The newly generated sequencing data used for all analyses in this paper have been deposited in the Genome Sequence Archive in the National Genomics Data Center, China National Center for Bioinformation/Beijing Institute of Genomics, Chinese Academy of Sciences (GSA‐Human: HRA005473 for discovery cohort; GSA: CRA015134 for validation cohort). Data supporting the findings are available within the paper and additional files. All other data related to the findings are also available from the corresponding author upon request. All the tools used for the data analysis are publicly available, and the version and parameters used have been indicated. All code generated in this study has been deposited on GitHub (https://github.com/Biofarmer/ELGR).
