# Supplementary material for: The Gut Resistome Atlas in Preterm Infants Enables Prediction of Necrotizing Enterocolitis Onset
Source: Adv Sci (Weinh). 2025 Sep 30;12(45):e05154. doi: 10.1002/advs.202505154 (PMC12677589; doi:10.1002/advs.202505154)
Supplement: Supplementary file 1 — Supporting Information [file ADVS-12-e05154-s007.pdf]

Supporting Information: The Gut Resistome Atlas in Preterm Infants Enables Prediction of Necrotizing Enterocolitis Onset

S. Zeng et al.

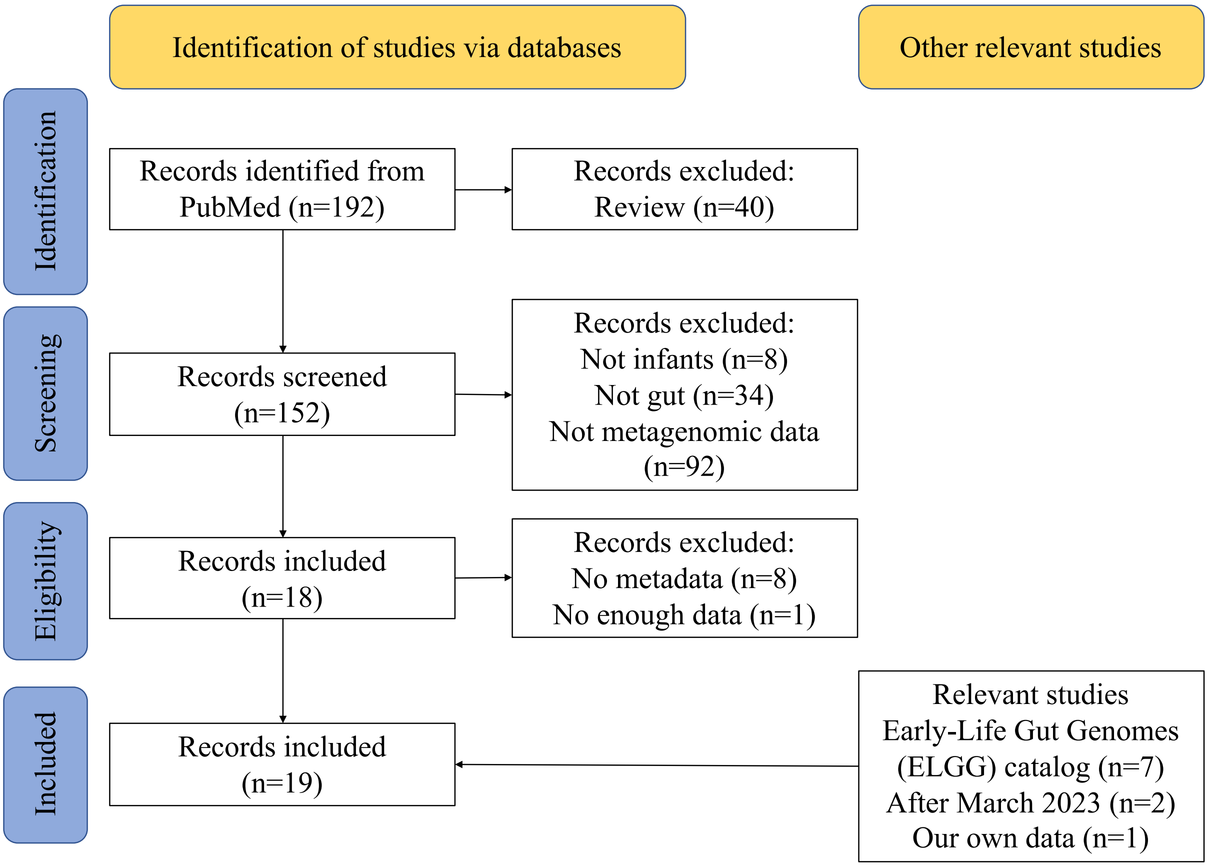

Figure S1 PRISMA flow diagram of the literature search

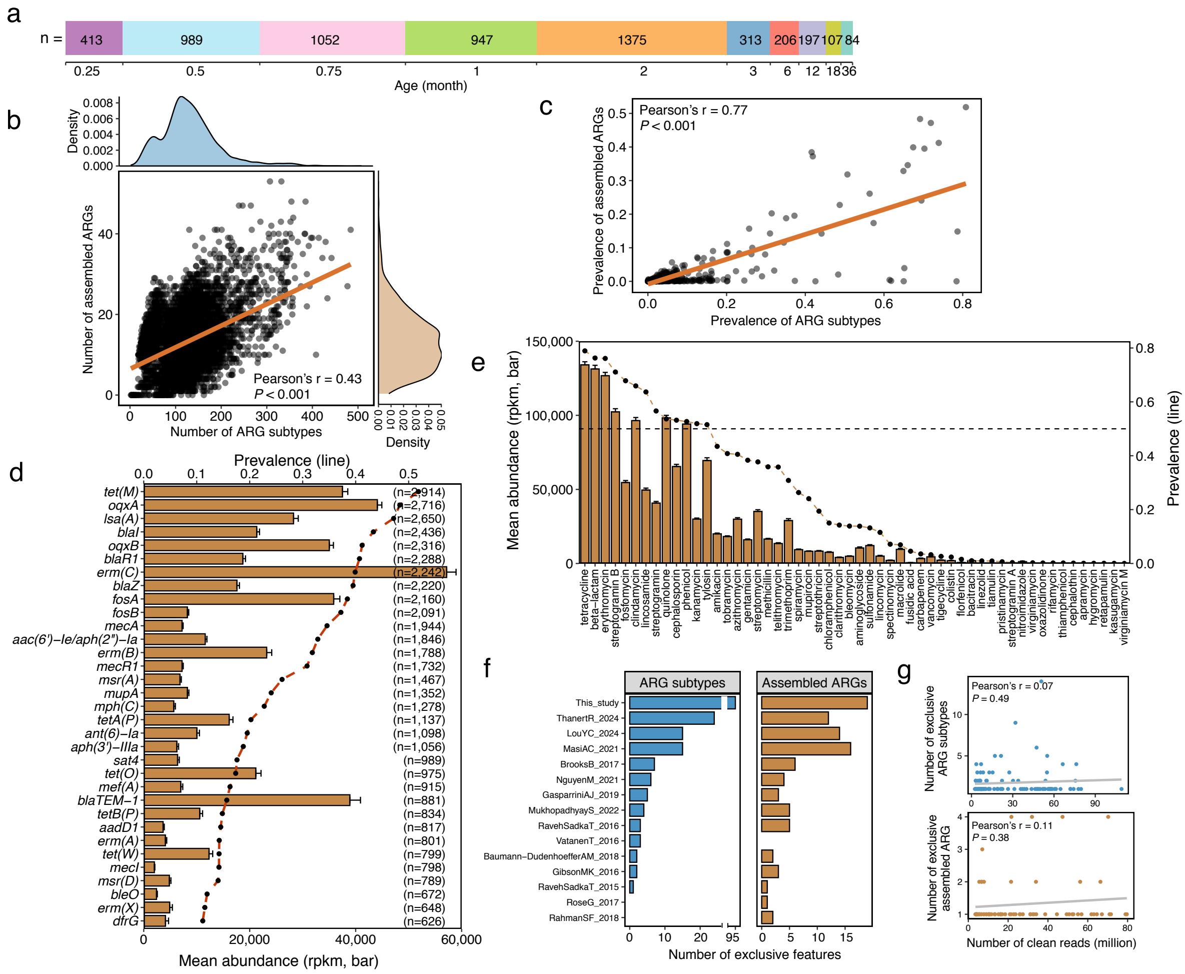

Figure S2 Characterization of gut resistome in preterm infants. a, Number of stool samples in each of timepoints. **b**, Significant correlation between number of ARG subtypes (read-based) and assembled ARGs (assembly-based) on a per-sample basis. The density plot on the top and right indicate the distribution of number of ARG subtypes and assembled ARGs in each sample, respectively. **c**, Significant correlation between prevalence of ARGs that were detected by both read-based and assembly-based approaches. **d**, Top aARGs with a prevalence > 10% detected by assembly-based approach in preterm infants. The red line indicates the prevalence of aARGs, and the bar indicates the mean abundance of aARGs across samples. The number in parenthesis indicates the number of samples having the corresponding aARGs. All the values in the bar plots are presented as mean ± SE. **f**, The prevalence (line) and abundance (bar) of ARG drug classes across samples from assembly-based approach. **g**, Number of exclusive ARG subtypes (left) and aARGs (right) in each cohort. All the values in the bar plots are presented as mean ± SE. **h**, Correlations of number of exclusive ARG subtypes (top) or aARGs (bottom) with sequencing depth on a per-sample basis.

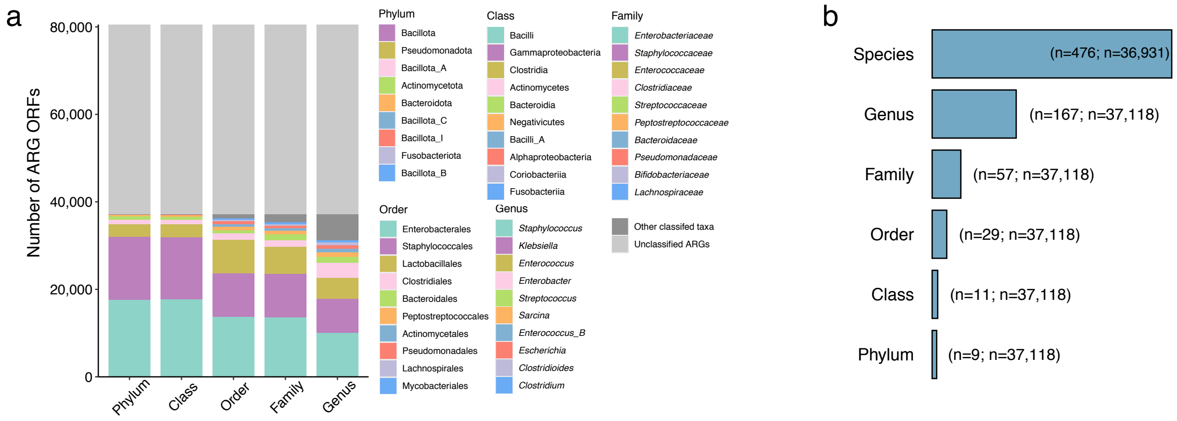

Figure S3 Taxonomic assignment of ARG ORFs in preterm infants. a, Taxonomic affiliation of ARG ORFs at different resolutions. Data is partitioned by known taxonomic rank, with only the 10 most highly represented known taxa of each rank depicted in the legend, and the other known taxa are annotated as 'Other classified taxa'. The ARGs without assignment of taxa are annotated as 'Unclassified ARGs'. **b**, The number of known taxa per rank (the left number in parenthesis) and ARG ORFs belonging to that rank (the right number in parenthesis).

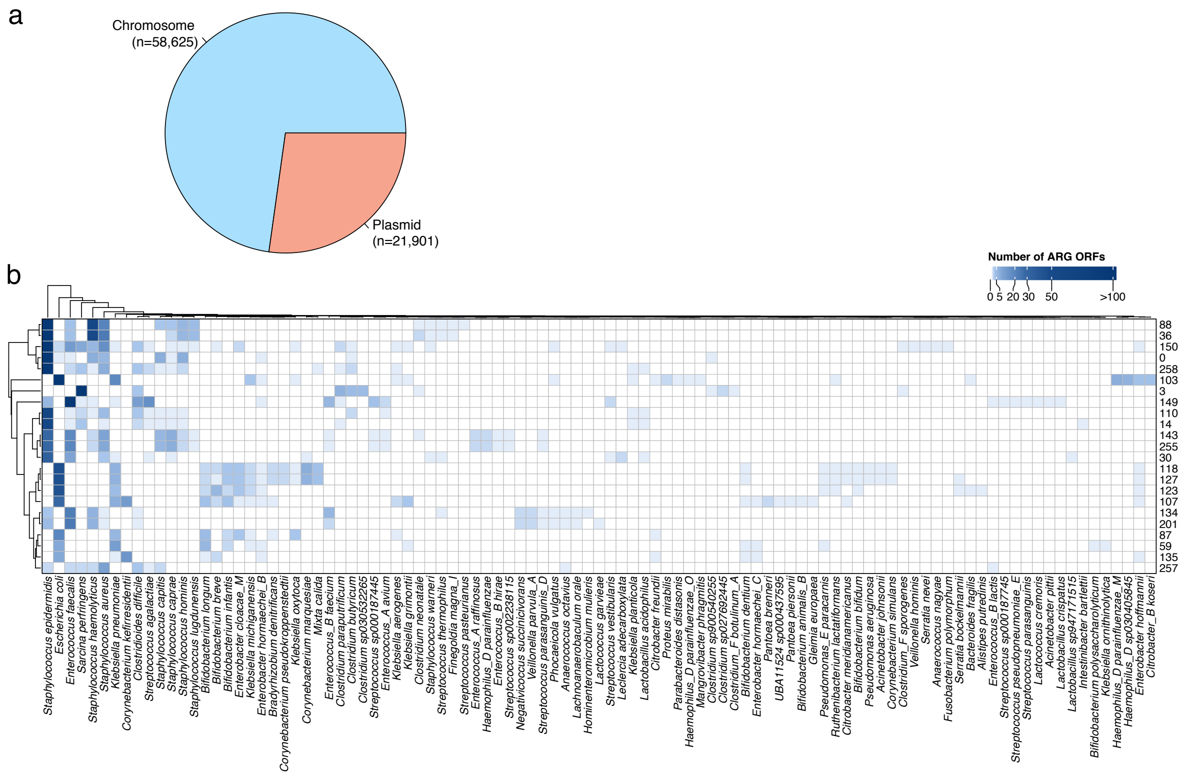

Figure S4 Genomic location of ARGs in preterm infants. **a**, Proportion of ARG ORFs from chromosome or plasmid. **b**, Distribution of ARG ORFs among different species in preterm infants. Only clusters containing ≥ 10 species are plotted. The color gradient indicates the number of ORFs from the species of the corresponding column.

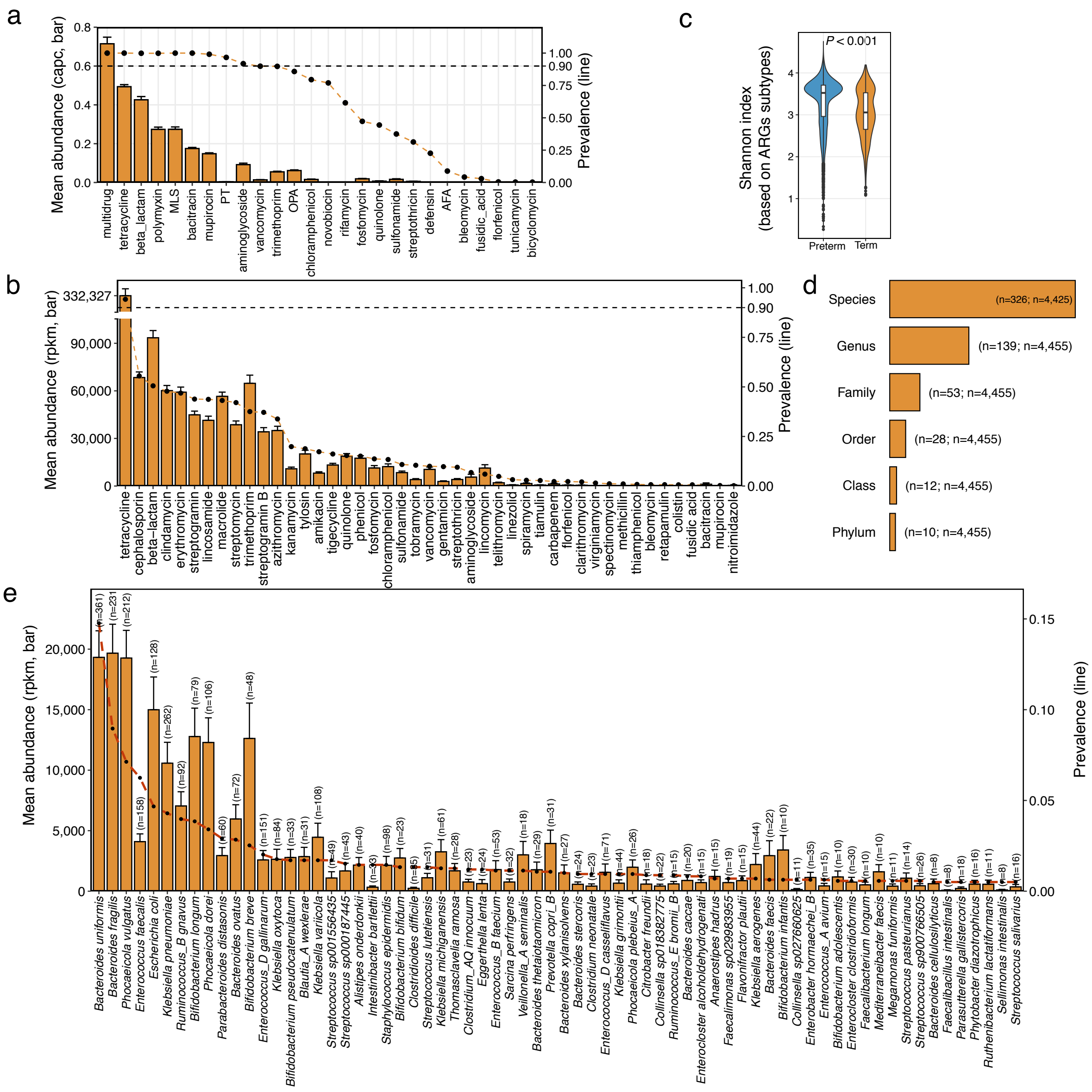

Figure S5 Composition of gut resistome in term infants. a, Prevalence (line) and mean abundance (bar) of ARG types across all samples. AFA = antibacterial fatty acid, MLS = macrolide-lincosamide-streptogramin, PT = pleuromutilin-tiamulin, OPA = other peptide antibiotics. All the values in the bar plots are presented as mean ± SE. **b**, Prevalence (line) and abundance (bar) of ARG drug classes from assembly-based approach. All the values in the bar plots are presented as mean ± SE. **c**, Shannon index of ARG subtypes in preterm infants was higher (linear mixed-effect model, $P < 0.001$) than that of term infants. **d**, Number of known taxa per rank (the left number in parenthesis) and ARG ORFs belonging to that rank (the right number in parenthesis). **e**, Prevalence (line) and mean abundance (bar) of ARG bacterial carriers at the species level based on the assembly approach. Only species with a prevalence > 0.5% across samples are plotted, and the number in parenthesis indicates the number of ARG ORFs harbored by that species. The values in the bar plots indicate the mean abundance of ARG ORFs carried by the corresponding species. All the values in the bar plots are presented as mean ± SE.

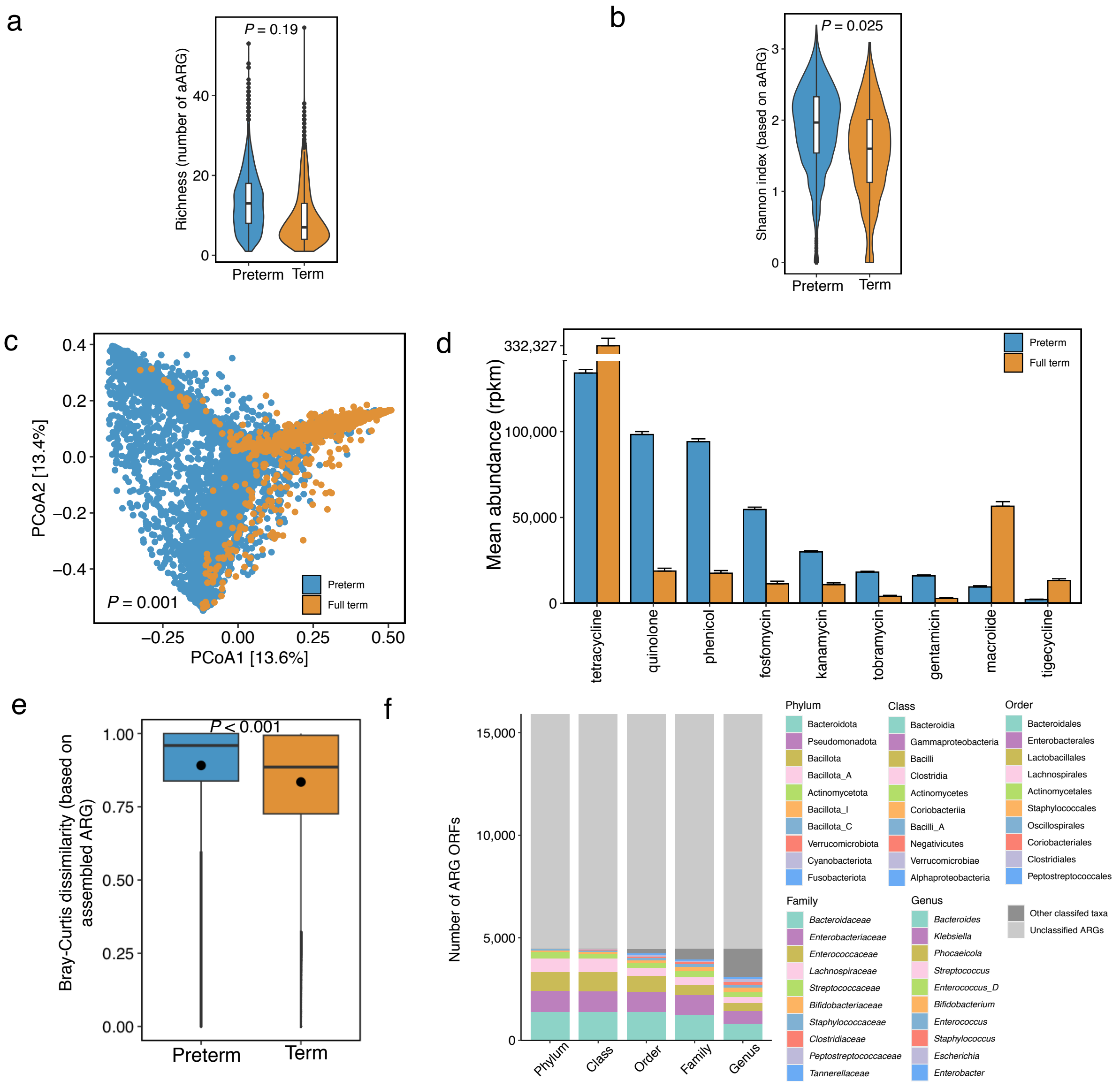

Figure S6 Comparisons of gut resistome between preterm and term infants based on assembly approach. The alpha diversity of aARGs in preterm infants was higher (**a**) for richness, (**b**) for Shannon index (linear mixed-effect model, $P < 0.05$) than that of term infants. **c**, Principal coordinate analysis (PCoA) based on Bray-Curtis distances of abundances of ARG families from all samples (PERMANOVA, $P = 0.001$). **d**, Preterm infants harboring higher abundances (linear mixed-effect model, FDR < 0.05) of six ARG drug classes and lower abundance of three ARG drug classes than that of term infants. Only aARGs with significant differences (FDR < 0.05) between preterm and term infants are plotted. All the values in the bar plots are presented as mean ± SE. **e**, Mean Bray-Curtis distances calculated from ARG families between samples from preterm infants was lower (two-sided Wilcoxon rank-sum test, $P < 0.001$) than that of full-term infants. The box plots show the interquartile range (IQR), with horizonal line as the median, points in the box as the mean, whiskers as the range of the data (up to 1.5× IQR), and points beyond the whiskers as outliers. **f**, Taxonomic affiliation of ARG carriers in term infants. Data is partitioned by known taxonomic rank, with only the 10 most highly

represented known taxa of each rank depicted in the legend, and the other known taxa are annotated as 'Other classified taxa'. The ARG ORFs without assignment of taxa are annotated as 'Unclassified ARGs'.

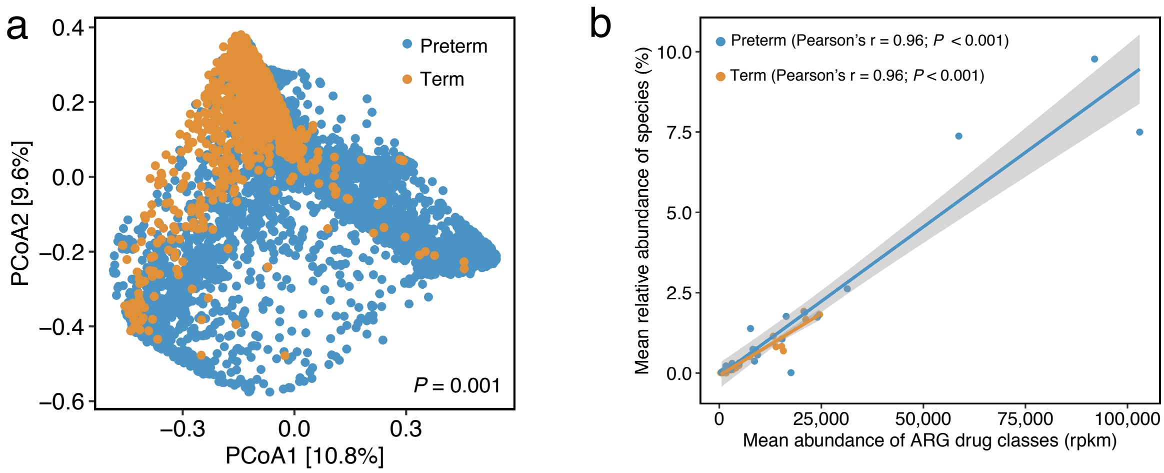

Figure S7 **a**, Principal coordinate analysis (PCoA) based on Bray-Curtis distances of abundance of bacterial species from preterm and term infants (PERMANOVA, $P = 0.001$). **b**, Significant correlation (Pearson's r = 0.96 for preterm or term infants; $P < 0.001$) between the mean abundances of ARG drug classes and bacterial species.

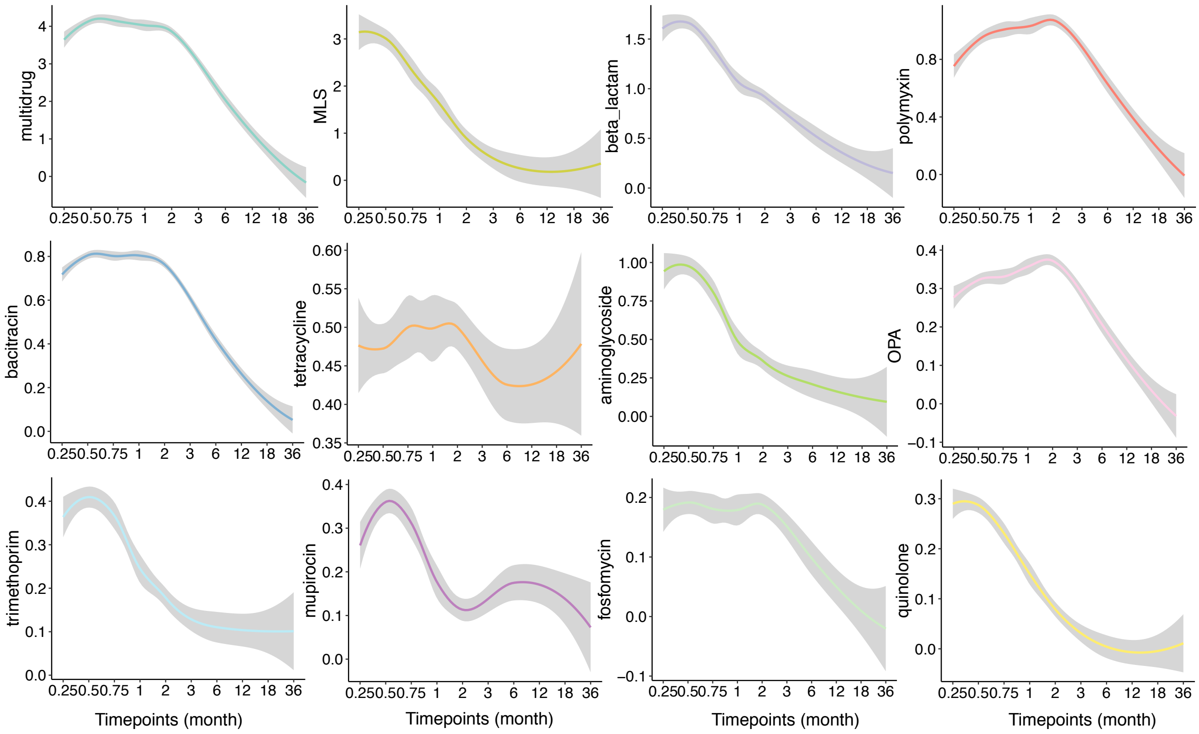

Figure S8 Dynamic changes of abundance of ARG types in the first three years of life from preterm infants. The Y axis indicates the abundance of ARG types, and the X axis is the age of infants stratified into 10 timepoints.

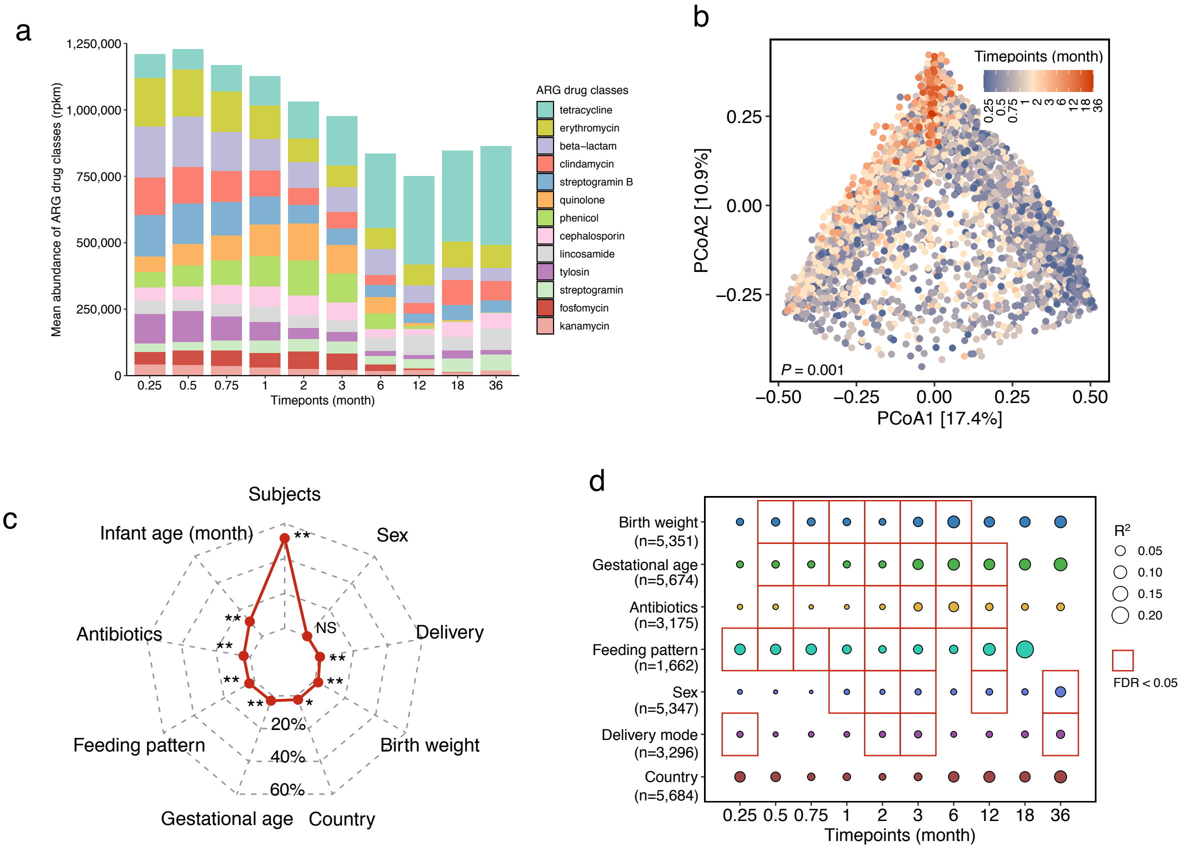

Figure S9 Dynamic composition of gut resistome and covariates in preterm infants based on aARGs from assembly approach. a, Changes of abundance of ARG drug classes in preterm infants. Only drug classes with a prevalence > 50% are plotted. **b**, Principal coordinate analysis (PCoA) ordination of gut resistome beta diversity measured by Bray–Curtis distances of aARGs abundance profiles per sample (n = 5,618). **c-d**, Significance and explained variance of seven clinical covariates when pooling all sample together (**c**) or stratified into distinct timepoints (**d**), determined by PERMANOVA on between-sample Bray–Curtis distances of aARGs. $^{*}P < 0.05$, $^{**}P < 0.01$.

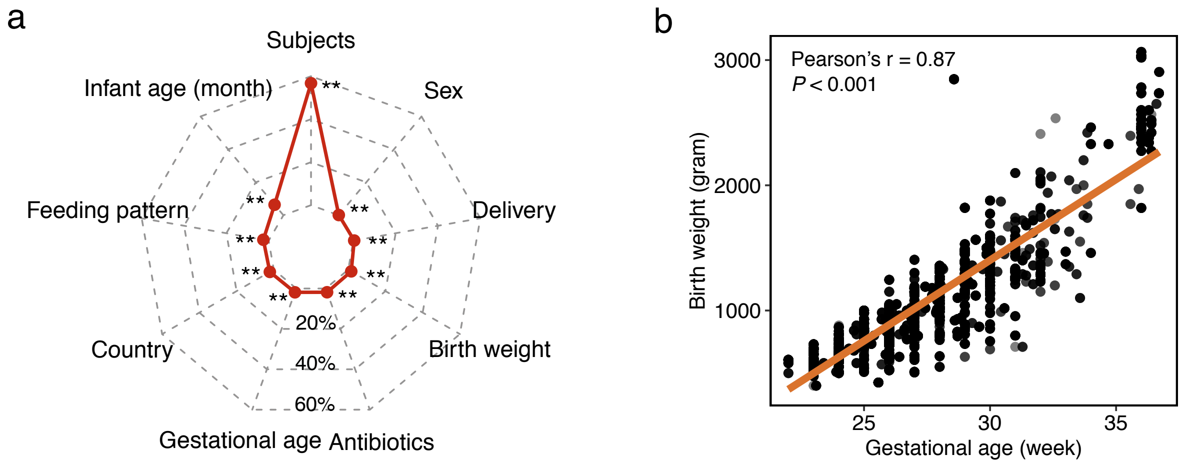

Figure S10 a, Significance and explained variance of seven clinical covariates when pooling all sample together determined by PERMANOVA on between-sample Bray–Curtis distances of ARG subtypes. $^{*}P < 0.05$, $^{**}P < 0.01$. **b**, Significant correlation between birth weight and gestational age.

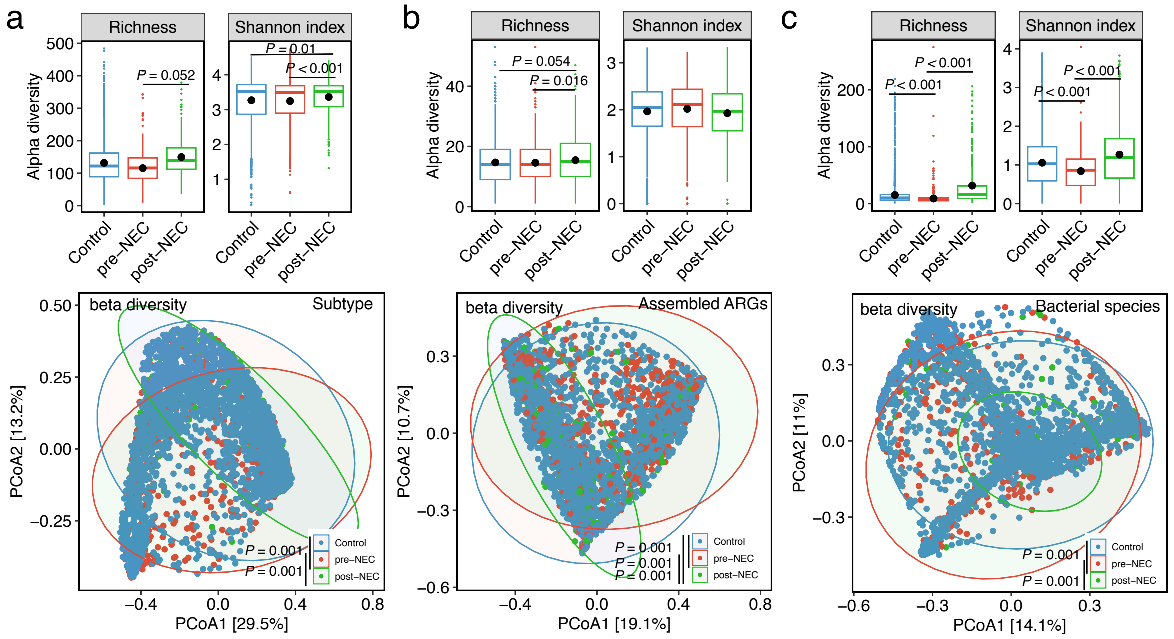

Figure S11 Comparisons of diversity of gut resistome between infants with or without NEC. a-c, Differences of alpha and beta diversity of ARG subtypes (**a**), assembled ARGs (**b**), and bacterial species (**c**) between infants with or without (control) NEC onset. The P values for alpha diversity were obtained from linear mixed-effect model, and beta diversity from PERMANOVA. The box plots show the interquartile range (IQR), with horizonal line as the median, points in the box as the mean, whiskers as the range of the data (up to 1.5× IQR), and points beyond the whiskers as outliers.

Supplemental Table legends

Table S1. The metadata of preterm infants included in this study.

Table S2. The summarized type, abundance and prevalence of ARG types and subtypes in preterm infants.

Table S3. The information of 80,526 ARG ORFs obtained from assembly-based approach of preterm infants, including assembled ARG names and drug classes to which ARGs belong, abundance, the taxonomic assignment and prediction of its origin.

Table S4. The summarized type, abundance and prevalence of assembled ARGs and drug classes in preterm infants.

Table S5. Clusters of plasmid-borne ARG ORFs with taxonomic assignment at the species level from preterm or term infants.

Table S6. The metadata of term infants included in this study for the gut resistome comparisons.

Table S7. The summarized type, abundance and prevalence of ARG types and subtypes in term infants.

Table S8. The information of 15,914 ARG ORFs obtained from assembly-based approach of term infants, including assembled ARG names and drug classes to which ARGs belong, abundance, the taxonomic assignment and prediction of its origin.

Table S9. Comparisons of ARG subtypes (with a prevalence > 5% either in preterm or term infants) between preterm and term infants.

Table S10. The metadata of studies with preterm infants with NEC onset, as well as the metadata for validation cohort.

Table S11. The selected stool samples and metadata of preterm infants in each window for identification of the accelerated convergence of gut gut resistome preceding NEC onset.

Table S12. Importance of features from random forest for the external validation.

Table S13. The data in accordance with the 'Strengthening The Organization and Reporting of Microbiome Studies' (STORMS) guidelines for the human microbiome research.
